# Bioinformatic analysis of the regulatory potential of tagging SNPs provides evidence of the involvement of genes encoding the heat-resistant obscure (Hero) proteins in the pathogenesis of cardiovascular diseases

**DOI:** 10.1515/jib-2024-0043

**Published:** 2025-06-03

**Authors:** Vladislav V. Shilenok, Irina V. Shilenok, Vladislav O. Soldatov, Yuriy L. Orlov, Ksenia A. Kobzeva, Alexey V. Deykin, Olga Yu Bushueva

**Affiliations:** Laboratory of Genomic Research, Research Institute for Genetic and Molecular Epidemiology, Kursk State Medical University, Kursk 305041, Russia; Division of Neurology, Kursk Emergency Hospital, 305035 Kursk, Russia; Joint Center for Genetic Technologies and Department of Pharmacology and Clinical Pharmacology, Belgorod State National Research University, Belgorod 308015, Russia; Center of Biodesign and Complex Systems Modeling, Sechenov First Moscow State Medical University (Sechenov University), Moscow 119991, Russia; Agrarian and Technological Institute, Peoples’ Friendship University of Russia, Moscow 117198, Russia; Department of Biology, Medical Genetics and Ecology, Kursk State Medical University, Kursk 305041, Russia; Cardiology Department with Intensive Care Unit, Kursk Emergency Hospital, 305035 Kursk, Russia

**Keywords:** Hero (heat-resistant obscure) proteins, cardiovascular diseases, QTL, histone modifications, transcription factors

## Abstract

Although multiple aspects of molecular pathology underlying cardiovascular diseases (CVDs) have been revealed, the complete picture has yet to be elucidated. In this respect, annotation of the novel links between genes and atherosclerosis is of great importance for cardiovascular medicine. Aligning with our previous research, we aimed to analyze the cardiovascular predisposition contribution of the genes encoding Hero-proteins, polypeptides with chaperone activity. Following bioinformatic sources were utilized to annotate data regarding the cardiovascular contribution of Hero-proteins and their genes: SNPinfo Web Server, The Cardiovascular Disease Knowledge Portal, GTEx Portal, HaploReg, rSNPBase, RegulomeDB, atSNP, Gene Ontology, QTLbase, and the Blood eQTL browser. Almost all analyzed genes were characterized by a very high regulatory potential of tag SNPs (except *BEX3*). Multiple substantial impacts of the analyzed SNPs on histone modifications, eQTL effects on CVD-related genes, and binding to transcription factors involved in biological processes pathogenetically significant for CVDs have been discovered. Here we provide *in silico* evidence of the involvement of genes *C9orf16 (BBLN)*, *C11orf58*, *SERBP1*, *SERF2*, and *C19orf53* in CVDs and their risk factors (high blood pressure, dyslipidemia, obesity, arrhythmias, etc.), thus revealing Hero-proteins as putative actors in the pathobiology of the heart and vessels.

## 1 Introduction

Cardiovascular diseases (CVDs) cause the greatest part of morbidity and mortality in the world. According to the World Heart Federation report, CVDs claim more than 20 million lives annually [1]. CVDs is an umbrella term, referring to any pathologies of the heart and blood vessels; however, the largest proportion of the burden is constituted by coronary heart disease [2]. Preferentially, it is related to the atherosclerotic lesions of vessels. Another significant contributor to mortality and long-term disability is hypertension [3], [4], [5]. About half of the cases of coronary heart disease can be attributed to high blood pressure [6].

The cellular and molecular mechanisms underlying the development of atherosclerosis and hypertension are apparently mostly based on a combination of altered lipid metabolism [7], abnormal inflammatory signaling [8], unbalanced humoral regulation [9], and dysfunction of endothelial cells [10]. Gene expression regulation by protein chaperone activity presents a novel point of view for CVD risk. Each mechanism is a result of the complex interaction of a large variety of modifiable risk factors, such as inappropriate diet or smoking, and non-modifiable (age, gender, genetics) risk factors [11]. However, those multifactorial mechanisms and risk factors are gradually being explained in more detail, disclosing novel genetic [12], [13], [14], environmental, and behavioral [15] correlates of the disease’s components.

Further findings that explain the pathobiology of CVDs are extremely helpful in the fight against the diseases. Some preliminary data concerning new factors that play a role in CVDs can be obtained with the use of the results of genome population-based studies. Analysis of SNPs is a powerful tool to answer whether a gene is involved in a given pathology and further target the associated pathway in detail. In our previous studies, we revealed an association between the risk of ischemic stroke and the genes *C9orf16*, *C19orf53, SERBP1,* and *SERF2* using a population genetics-based approach [16], [17], [18], [19]. These genes encode proteins belonging to the recently discovered family of Hero (heat-resistant obscure) proteins. These proteins, along with BEX3 and C11orf58, display very high chaperone-like activity *in vitro* and *in vivo* [20], with a high protective effect against pathological protein aggregation, particularly of DNA-binding protein 43 (TDP-43) [21]. Moreover, other studies have revealed their involvement in different pathological processes. For instance, increased expression of *C9orf16* (*BBLN)* was observed in primary and metastatic cancer cells of pancreatic ductal adenocarcinoma (PDAC) cells [22], a significant role of this gene in ovarian cancer progression was noted [23]. Overexpression of the *BEX3* gene has been linked to cisplatin chemoresistance in nasopharyngeal carcinoma [24] and growth control of F9 teratocarcinoma cells [25]. The *C11orf58* gene is associated with the risk of immune infiltration and progression in melanoma [26]. *C9orf16* (*BBLN*) has been significantly regulated in synovitis of osteoarthritis [27], osteoporosis through its influence on bone mineral density [28]. BEX3 affects skull morphology, neuron populations, and hippocampal balance, which may explain certain behavioral changes, including schizophrenia and autism [29].

Here, we aimed to perform a bioinformatic analysis of the overall contribution of the tag SNPs of genes *C11orf58* (Hero-20), *C19orf53* (Hero-11), *C9orf16* (Hero-9), *C19orf53* (Hero-11), *SERBP1* (Hero-45), and *SERF2* (Hero-7), encoding the Hero-proteins to cardiovascular diseases. We discuss the mechanisms of Hero-proteins action in the pathobiology of the heart and vessels.

## 2 Materials and methods

**Figure 1:**
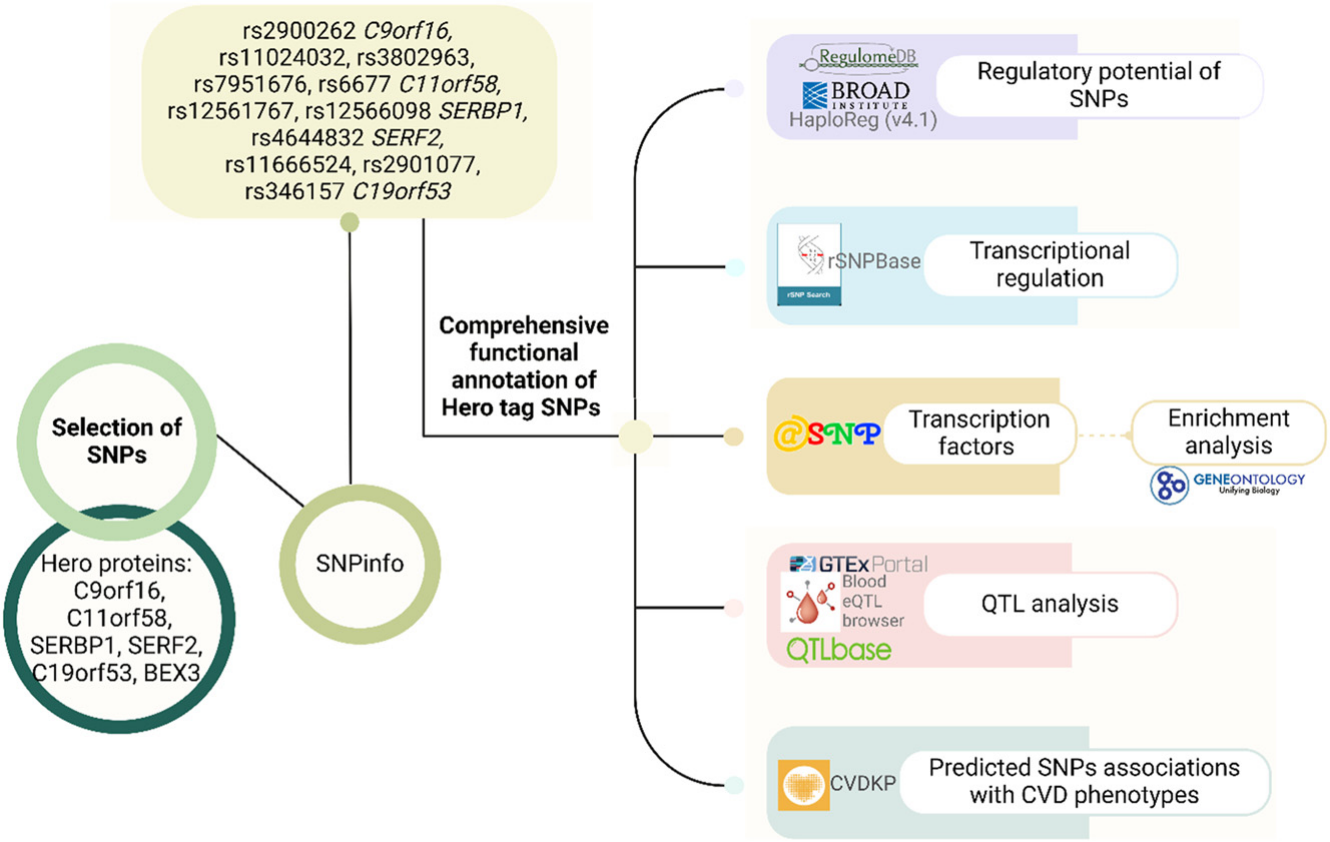
Study overview and methodology.

The study overview and methodology are outlined in Figure 1. The study included genes encoding heat-resistant obscure (Hero) proteins [20]. All Hero genes are expressed in blood vessels, heart tissue, and peripheral blood, which makes them attractive for research in the field of cardiovascular pathology (Supplementary Figure 1A–F).

### 2.1 Selection of polymorphisms

For maximum coverage of the Hero gene structure, tagging SNPs (tag SNPs) representing a group of linked polymorphisms (haplotypes) inherited with linkage equilibrium (D’≥0.8) were selected. These tag SNPs are used as informative surrogates for all common variants in the human genome. In particular, SNP arrays constructed using “tag SNP” are also used to conduct genome-wide association studies (GWAS) 57. In this regard, we also focused on tag SNPs in our study.

The bioinformatics tool SNPinfo Web Server [30] was used to select SNPs based on the reference haplotype structure of Caucasian populations (CEU) of the HapMap project. One member of the Hero-proteins family, BEX3, was not included in our analysis since its gene does not contain tag SNPs. All other Hero genes are characterized by the presence of tag SNPs, and the identified tag SNPs have a frequency of at least one of the minor alleles (MAF) in the European population of at least 5 %, which makes them valuable for the study of multifactorial human diseases. Thus, a total of 11 tag SNPs were selected in 5 Hero genes: rs2900262 in the *C9orf16* (*BBLN*) gene*,* rs11024032, rs3802963, rs7951676, rs6677 in the *C11orf58* gene*,* rs12561767, rs12566098 in the *SERBP1* gene*,* rs4644832 in the *SERF2* gene*,* rs11666524, rs2901077, rs346157 in the *C19orf53* gene. Ten tagging SNPs are localized in introns, and one (rs6677 *C11orf58*) is located in the 3′-UTR of the gene.

### 2.2 Bioinformatic analysis

In total 11 bioinformatics tools were employed for the analyses.

SNPinfo Web Server–SNP Function Prediction [30] was employed to preliminarilyassess the regulatory potential of SNPs (Regulatory Potential Score) [31].

The bioinformatics source HaploReg (v4.2) [32] was utilized to evaluate the association of SNPs with histone modifications marking promoters and enhancers: monomethylation of the 4th lysine residue on histone H3 protein (H3K4me1), trimethylation of the 4th lysine residue on histone H3 protein (H3K4me3), acetylation of the 9th lysine residue on histone H3 protein (H3K9ac), acetylation of the 27th lysine residue on histone H3 protein (H3K27ac). This resource was also used to analyze the localization of SNPs in regions of DNase hypersensitivity, regions of regulatory motifs, and binding sites with regulatory proteins [33].

The rSNPBase tool was employed to analyze the effects of SNPs on proximal transcriptional regulation, distal transcriptional regulation, RNA-binding protein-mediated regulation, and microRNA-mediated regulation [34].

RegulomeDB (Version 1.1) [35] was utilized to estimate the regulatory coefficients of SNPs [36].

The atSNP bioinformation database [37] (was used to analyze the DNA binding sites with transcription factors (TFs) depending on the carriage of the reference/alternative alleles [38].

The bioinformatics tool Gene Ontology [39] was employed to analyze the overrepresented biological processes characterizing transcription factors binding to the reference/alternative alleles [40].

QTLbase [41] was utilized to analyze the binding of SNPs to methylation quantitative trait loci (mQTL) in peripheral blood, heart, aorta/arteries of various locations [42].

To analyze the eQTL effects of tag SNPs of Hero genes in peripheral blood, the Blood eQTL browser resource [43] was used [44].

GTEx portal [45] was employed to analyze the expression levels of the studied genes in whole blood, blood vessels, heart as well as to analyze the association of SNPs with expression quantitative trait loci (eQTL). The expression significance of SNPs (*cis*-eQTL) was studied in peripheral blood, aorta/arteries of various locations [46].

The Cardiovascular Disease Knowledge Portal (CVDKP) [47] was utilized for bioinformatics analysis of associations of the studied tag SNPs with other cardiovascular diseases of atherosclerotic origin and risk factors (such as blood pressure, body mass index, LDL, HDL level, etc.) CVDKP is part of the Common Metabolic Diseases Knowledge Portal (CMDKP) project, which aggregates and analyzes genetic association results across multiple populations, including mixed ancestry cohorts. It integrates data from the tAtrial Fibrillation Consortium (AFGen), the Global Lipids Genetics Consortium (GLGC), the Heart Failure Molecular Epidemiology for Therapeutic Targets (HERMES), The Myocardial Infarction Genetics Consortium (MIGen), the CARDIoGRAMplusC4D Consortium and displays results of computational prediction methods to provide data, visualizations, and tools in an open-access portal [48].

## 3 Results

According to preliminary analysis using bioinformatics sources, all tag SNPs in Hero genes exhibit significant regulatory potential. The rSNPBase source revealed that all studied tagged SNPs differed in distal transcriptional regulation, as well as regulation mediated by RNA-binding proteins. Proximal transcriptional regulation was found for all SNPs except for rs2900262 in the *C9orf16 (*also known as *BBLN)* gene; however, the latter differs in microRNA-mediated regulation. According to RegulomeDB, most of the studied tag SNPs are characterized by regulatory coefficients of 4 – TF binding + DNase peak (rs3802963, rs12566098, rs4644832, rs11666524, rs346157) and 5 – TF binding or DNase peak (rs11024032, rs6677, rs2901077). Moreover, rs2900262 is characterized by a regulatory coefficient of 1b (eQTL + TF binding + any motif + DNase Footprint + DNase peak), and rs12561767 of 1f (eQTL + TF binding/DNase peak). The bioinformatics source HaploReg (v4.1) showed that the studied SNPs were characterized by histone modifications in the enhancer/promoter region, regions of hypersensitivity to DNase-1 in various tissues, binding sites with regulatory proteins, altered by DNA regulatory motifs. According to SNPinfo Web Server: SNP Function Prediction, rs7951676 in the *C11orf58* gene and rs4644832 in the *SERF2* gene are characterized by regulatory coefficients of 0.095 and 0.403, respectively (Supplementary Table 1).

### 3.1 Functional annotation of tag SNPs of hero gene

#### 3.1.1 QTL-effects

According to the GTEx Portal data, the tag SNPs of the studied genes were characterized by a *cis*-eQTL-mediated effect on the expression level of specific Hero genes (*SERBP1*, *SERF2*, *C19orf53*), together with several other genes (*IL12RB2*, *AC011330.5*, *ADAL*, *CATSPER2*, *CATSPER2P1*, *HYPK*, *MAP1A*, *STRC*, *STRCP1*, *ZSCAN29*, *MRI1*) within the cardiovascular system (Table 1).

**Table 1:** Effect of tag SNPs of genes *C11orf58 and C19orf53* on gene expression in peripheral blood (according to the Blood eQTL browser) and in tissues of the cardiovascular system through cis-eQTL effects (according to the GTEx Portal browser).

	Blood eQTL browser					GTEx portal browser				
SNP	Effect allele	Gene expressed	Z-score	P-value	FDR	Effect allele	Gene expressed	P-value	Effect (NES)	Tissue
rs2900262 *C9orf16 (*T/C)	T	*URM1*	−10.688	1.15×10^−26^	0					
		*RP11-395P17.3*	5.781	7.41×10^−9^	4.52×10^−5^					
		*DNM1*	−4.675	2.94×10^−6^	0.008					
		*SH3GLB2*	4.445	8.80×10^−6^	0.028					
rs11024032 *C11orf58* (C/T)	T	*C11orf58*	−12.242	1.85×10^−34^	0	–	–	–	–	–
		*PIK3C2A*	−4.332	1.48×10^−5^	0.038					
rs3802963 *C11orf58* (C/G)	C	*SOX6*	5.921	3.20×10^−9^	2.6×10^−5^	–	–	–	–	–
rs7951676 *C11orf58* (G/T)	T	*C11orf58*	−8.212	2.17×10^−16^	0	–	–	–	–	–
rs6677 *C11orf58* (T/G)	G	*C11orf58*	18.480	3.01×10^−76^	0	–	–	–	–	
rs4644832 *SERF2* (G/A)	G	*ZSCAN29*	−46.525	3.27×10^−310^	0	A	*AC011330.5*	2.2×10^−7^	↑(0.52)	Heart–atrial appendage
		*SERF2*	−26.111	2.72×10^−150^	0		*AC011330.5*	4.4×10^−7^	↑(0.45)	Artery–aorta
		*TUBGCP4*	−23.763	8.03×10^−125^	0		*AC011330.5*	1.1×10^−5^	↑(0.29)	Artery–tibial
		*STRCP1*	20.637	1.28×10^−94^	0		*AC011330.5*	3.7×10^−5^	↑(0.50)	Artery–coronary
		*LCMT2*	18.252	2.01×10^−74^	0		*ADAL*	7.6×10^−6^	↓(−0.24)	Artery–tibial
		*CATSPER2*	11.870	1.70×10^−32^	0		*CATSPER2*	4.0×10^−6^	↓(−0.21)	Artery–tibial
		*PDIA3*	−10.794	3.65×10^−27^	0		*CATSPER2P1*	1.3×10^−5^	↓(−0.27)	Artery–tibial
		*MAP1A*	9.126	7.13×10^−20^	0		*MAP1A*	7.8×10^−5^	↓(−0.18)	Artery–aorta
		*STRC*	9.038	1.59×10^−19^	0					
		*CATSPER2P1*	8.223	1.99×10^−16^	0		*SERF2*	3.3×10^−7^	↑(0.17)	Artery–aorta
		*ADAL*	6.717	1.85×10^−11^	0		*SERF2*	7.9×10^−6^	↑(0.09)	Artery–tibial
		*TRIM69*	6.292	3.14×10^−10^	0		*STRC*	7.5×10^−6^	↓(−0.30)	Artery–aorta
							*STRC*	1.7×10^−5^	↓(−0.25)	Artery–tibial
							*STRCP1*	7.2×10^−7^	↓(−0.28)	Artery–tibial
							*STRCP1*	1.2×10^−6^	↓(−0.33)	Artery–aorta
							*HYPK*	8.5×10^−6^	↑(0.20)	Heart–left ventricle
							*ZSCAN29*	2.3×10^−5^	↑(0.20)	Heart–atrial appendage
rs12561767 *SERBP1* (G/A)	G	*IL12RB2*	26.197	2.92×10^−151^	0	A	*IL12RB2*	2.8×10^−10^	↑(0.28)	Artery–tibial
							*SERBP1*	2.3×10^−6^	↓(−0.09)	Artery–tibial
rs12566098 *SERBP1* (C/G)	C	*IL12RB2*	17.398	8.60×10^−68^	0	G	*SERBP1*	1.3×10^−9^	↓(−0.12)	Artery–tibial
							*IL12RB2*	2.3×10^−11^	↑(0.30)	Artery–tibial
rs11666524 *C19orf53* (G/A)	A	*MRI1*	−39.214	3.27×10^−310^	0	A	*C19orf53*	5.5×10^−18^	↓(−0.23)	Artery–aorta
		*CCDC130*	9.1379	6.37×10^−20^	0		*C19orf53*	1.7×10^−17^	↓(−0.17)	Artery–tibial
		*ZSWIM4*	6.544	5.99×10^−11^	0		*C19orf53*	8.7×10^−16^	↓(−0.42)	Brain–cortex
		*C19orf53*	−46.273	3.27×10^−310^	0		*C19orf53*	2.0×10^−7^	↓(−0.14)	Heart–atrial appendage
							*C19orf53*	7.9×10^−8^	↓(−0.14)	Heart–left ventricle
							*MRI1*	9.3×10^−6^	↓(−0.23)	Artery–tibial
							*MRI1*	2.1×10^−5^	↓(−0.27)	Artery–aorta
rs2901077 *C19orf53* (C/T)	T	*MRI1*	13.9143	5.19×10^−44^	0	–	–	–	–	–
		*C19orf53*	11.0932	1.36×10^−28^	0					
		*ZSWIM4*	9.657	4.59×10^−22^	0					
		*CCDC130*	−4.7835	1.72×10^−6^	0.005					
rs346157 *C19orf53* (A/G)	G	*C19orf53*	−31.2513	2.14×10^−214^	0	G	*C19orf53*	3.2×10^−10^	↓(−0.11)	Artery–tibial
		*MRI1*	−26.0647	9.14×10^−150^	0		*C19orf53*	6.2×10^−10^	↓(−0.15)	Artery–aorta
		*CCDC130*	12.596	2.22×10^−36^	0					

FDR, false discovery rate; NES, normalized effect size. Gene names are italicized.

Additionally, using the Blood eQTL browser, an analysis of the eQTL effects of tag SNPs of Hero genes in peripheral blood was carried out. It was revealed that tag SNPs of the studied genes not only affect the expression of *C11orf58, SERF2*, and *C19orf53* in peripheral blood but are also characterized by an eQTL effect on the expression level of the following genes: *ADAL*, *CATSPER2*, *CATSPER2P1*, *CCDC130*, *DNM1*, *IL12RB2*, *LCMT2*, *MAP1A*, *MRI1*, *PDIA3*, *PIK3C2A*, *RP11*-*395P17.3*, *SH3GLB2*, *SOX6*, *STRC*, *STRCP1*, *TRIM69*, *TUBGCP4*, *URM1*, *ZSCAN29*, *ZSWIM4* (Table 1).

Subsequent analysis of sQTL effects established that three of the studied SNPs of the Hero genes–rs4644832 in the *SERF2* gene, rs11666524, and rs346157 in the *C19orf53* gene–are characterized by associations with splicing quantitative trait loci, affecting the alternative splicing of the genes *AC011330*.*5*, *CATSPER2*, and *C19orf53* in blood vessels and heart (Supplementary Table 2).

Moreover, all tag SNPs of Hero genes (except for rs6677 *C11orf58* and rs11024032 *C11orf58)*, through their connection with mQTL, jointly affect the methylation level of 29 CpG sites (cg09976142, cg10071929, cg11884704, cg13518265, cg13588599, cg13642260, cg14140152, cg24392274, cg15378786, cg01977079, cg17284609, cg18749349, cg08660285, cg24364144, cg12861797, cg06158227, cg12032620, cg16445139, cg16487861, cg21033855, cg21245717, cg13587756, cg09254823, cg09952620, cg21192260, cg25722029, cg16474696, cg01530988, cg25755428) in blood cells (Supplementary Table 3).

#### 3.1.2 Histone modifications

Using the bioinformatics tool Haploreg, it was found that all tag SNPs of Hero genes are characterized by histone modifications; the majority of these SNPs demonstrate histone modifications in cardiovascular tissues and blood cells (Supplementary Table 4). Specifically, for rs11024032 in the *C11orf58* gene, hypersensitive sites have been identified for DNAse 1 in peripheral blood cells.

Moreover, using the resource HaploReg (v4.2), it was found that rs3802963 *C11orf58* is located in the binding site DNA with POL2 regulatory protein; rs12561767 *SERBP1* – with POL24H8 regulatory protein; rs4644832 *SERF2* – with 7 regulatory proteins: POL2, POL2B, POL24H8, TBP, YY1, GTF2B, HEY1.

#### 3.1.3 Transcription factors

**Figure 2:**
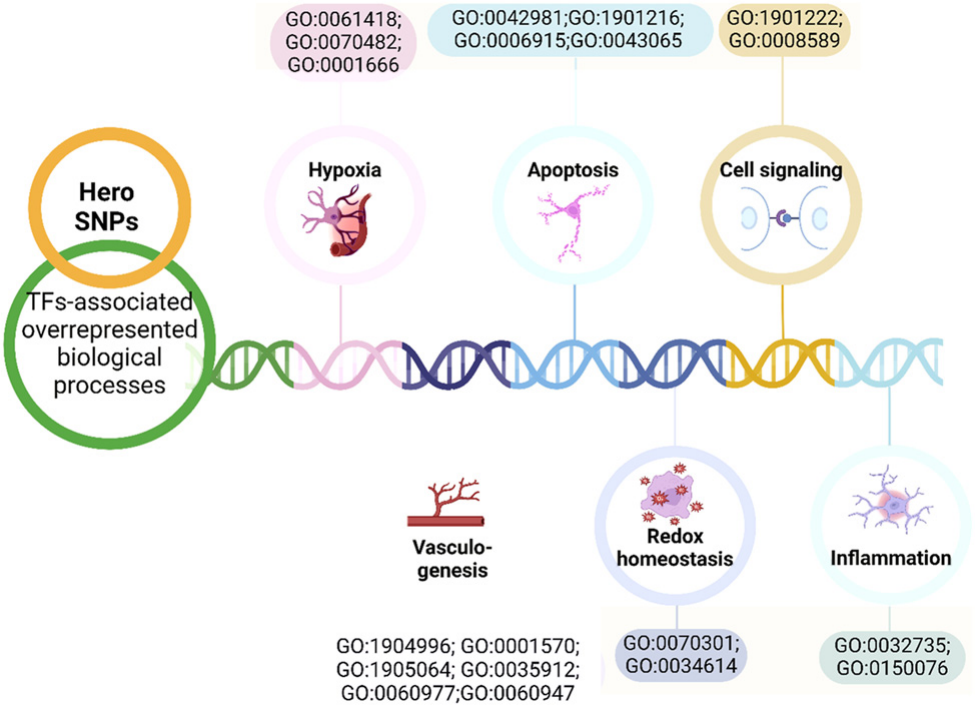
Overrepresented biological processes associated with transcription factors binding to the reference/alternative alleles.

Using the bioinformatics tool atSNP, it was established that tag SNPs of *SERF2*, *C11orf58,* and *C19orf53* Hero genes affect DNA binding to transcription factors depending on the carriage of the reference/alternative alleles (Figure 2, Supplementary Tables 5–12).

Notably, these transcription factors are potentially involved in processes crucial to the pathogenesis of cardiovascular pathology. For example, TFs binding to the alternative T allele of rs11024032 in the *C11orf58* gene jointly participate in the positive regulation of leukocyte adhesion to vascular endothelial cell (GO:1904996; FDR = 0.046); regulation of non-canonical NF-kappaB signal transduction (GO:1901222; FDR = 0.029) (Supplementary Table 4). TFs associated with the alternative allele G rs3802963 in the *C11orf58* gene participate in the regulation of apoptotic process (GO:0042981; FDR = 0.047). Meanwhile, TFs binding to the reference allele C are involved in the regulation of transcription from RNA polymerase II promoter in response to hypoxia (GO:0061418; FDR = 0.019) (Supplementary Table 6). TFs associated with the alternative allele G rs6677 in the *C11orf58* gene are implicated in coronary vasculature morphogenesis (GO:0060977; FDR=0.02), vasculogenesis (GO:0001570; FDR=0.007), whereas the reference allele T of rs6677 in the *C11orf58* gene is linked with TFs involved in negative regulation of vascular associated smooth muscle cell differentiation (GO:1905064; FDR = 0.033); positive regulation of leukocyte adhesion to vascular endothelial cell (GO:1904996; FDR = 0.0097); positive regulation of interleukin-12 production (GO:0032735; FDR = 0.038); cellular response to hydrogen peroxide (GO:0070301; FDR = 0.008); regulation of transcription from RNA polymerase II promoter in response to hypoxia (GO:0061418; FDR = 0.047); apoptotic process (GO:0006915; FDR = 0.02); positive regulation of apoptotic process (GO:0043065; FDR = 0.007) (Supplementary Table 7). The reference allele G SNP rs4644832 in the *SERF2* gene creates binding sites for TFs involved in the regulation of smoothened signaling pathway (GO:0008589; FDR = 0.02) (Supplementary Table 8). The reference allele C rs2901077 creates sites for DNA binding with transcription factors jointly involved in cardiac vascular smooth muscle cell differentiation (GO:0060947; FDR = 0.019); dorsal aorta morphogenesis (GO:0035912; FDR = 0.019); response to oxygen levels (GO:0070482; FDR = 0.035) (Supplementary Table 9). The SNP allele G rs346157 in the *C19orf53* gene is associated with TFs involved in positive regulation of cellular response to reactive oxygen species (GO:0034614; FDR = 0.035) and response to hypoxia (GO:0001666; FDR = 0.02) (Supplementary Table 10).

In addition, during the functional annotation of the *SERBP1* and *C9orf16* (*BBLN*) tag SNPs we previously studied, in the aspect of TF analysis, one more biological process was discovered that can play a significant role in CV pathology positive regulation of cytokine production (GO:0001819) [17], 19].

#### 3.1.4 Bioinformatic analysis of the association of tag SNPs in genes encoding hero proteins with cardio- and cerebrovascular diseases and CVD-related phenotypes

According to the bioinformatics resource Cardiovascular Disease Knowledge Portal (CVDKP), which combines and analyzes the results of genetic associations from the largest consortiums for the study of cardiovascular diseases, all Hero tag SNPs are characterized by phenotypic effects not only on common cardiovascular diseases (hypertension/blood pressure, coronary artery disease, myocardial infarction, and peripheral artery disease) but also on the risk factors, such as cholesterol and lipoproteins/apolipoproteins levels, body mass index, atrial fibrillation, increases in systolic blood pressure, and heart rate, which play a key role in predisposition to cardiovascular pathology.

## 4 Discussion

The main function of chaperones is to assist in the folding, refolding, and utilization of proteins [49]. However, chaperones play a pivotal role in a number of physiological processes as well as in responses to pathology [50], [51], [52], [53], [54], orchestrating inflammatory pathways [55], 56], response to oxidative stress [57], and many others [58]. Given their immense role in cellular functions, chaperones are considered dramatically important players in human pathology. In this regard, a growing body of research focuses on defects of the chaperone system as markers and drivers of CVDs [59], [60], [61], [62], [63].

Hero-are six proteins recently shown to preserve client proteins from proteotoxic stress by physicochemical interaction with them in a shield-like manner [20]. In brief, Tsuboyama et al. have revealed that C9orf16 (BBLN), C11orf58, BEX3, SERBP1, SERF2, and C19orf53 (Hero9, −20, −13, −45, −7, and −11, respectively) are highly charged, disordered, and heat-resistant proteins demonstrating chaperone-like activity *in vivo*. The authors have shown that Hero-proteins protect the activity of various proteins under stress conditions, prevent pathogenic neuronal protein aggregation *in vitro* and *in vivo*, and, finally, promote longevity in Drosophila. Recent *in silico* chemistry data also disclose some details of how Hero may interact with client proteins [21].

In order to address the involvement of Hero-proteins in the pathobiology of CVDs, we have previously conducted a genetic study exploring the associations of the genes *C19orf53, SERBP1*, *SERF2*, and *C9orf16* (*BBLN*) with ischemic stroke [16], [17], [18], [19]. Here, we report the high regulatory potential of Hero tag SNPs in coronary and peripheral arteries and heart tissues, suggesting their involvement in CVDs, providing ground for further research into their roles in this context [59].

First of all, analyzed genes are expressed in the vessels (aorta, coronary artery, tibial artery), heart (atrial appendage, left ventricle), and whole blood. Levels of their expression might be regulated via eQTLs related to studied tagSNPs: tagSNPs of genes *SERBP1, SERF2, C19orf53* are characterized by *cis*-eQTL-effects. Notably, the same tagSNPs also influence the expression of genes *IL12RB2, ADAL, CATSPER2, CATSPER2P1, HYPK, MAP1A, STRC, STRCP1, ZSCAN29, MRI1*, some of which are characterized by high pathogenetic significance for cardiovascular pathology.

For instance, the gene *MRI1* has been reported to be associated with cardiac pathology [64], 65]. Another *cis*-eQTL-linked gene, *ADAL* encodes Adenosine Deaminase-Like Protein, which is predicted to facilitate adenosine deaminase activity [66]. Serum adenosine deaminase activity is notably reduced in patients with CAD, particularly in cases of myocardial infarction [67]. *IL12RB2,* involved in IL-35 control, plays a crucial role in atherosclerosis and inflammation regulation [68]. It has also been found to inhibit ischemia/hypoxia-induced angiogenesis, suggesting that this anti-inflammatory cytokine plays new roles at the recovery stage of angiogenesis [69]. *HYPK*, acting as a regulator of the heat shock response – an important mechanism in other cardiovascular disorders [46]. STRC (stereocilin) expression is downregulated 1.5-fold in the blood of CAD patients, and it interacts with mesothelin [70]. *STRCP1* is predicted to be located in extracellular region and to be involved in cell-matrix adhesion [71].

The impact of tag SNPs on the expression levels of Hero genes could be modulated by their association with quantitative methylation trait loci. This is significant because changes in methylation levels can either increase or decrease gene expression [72]. We observed a correlation between the studied Hero genes tag SNPs (except for rs6677 and rs11024032 in the *C11orf58* gene) and the methylation levels of CpG sites via cis-mQTL effects in the blood and cardiovascular system. This underscores a pronounced tissue-specific epigenetic regulation of Hero, which holds considerable relevance for understanding their involvement in the mechanisms of CVDs.

**Figure 3:**
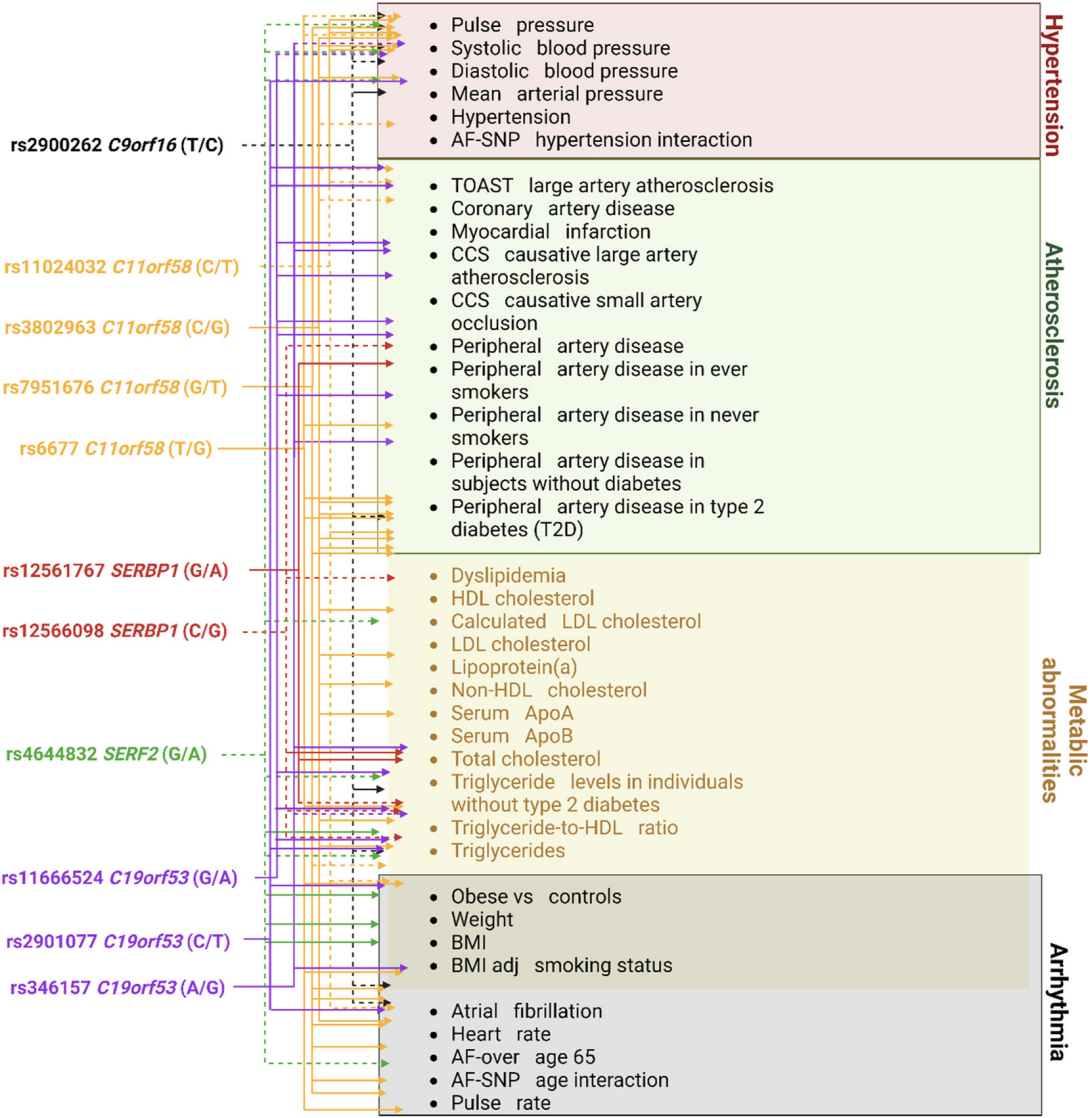
Associations of tag SNPs of the genes encoding Hero-proteins with certain cardiovascular phenotypes based on data extracted from the cardiovascular disease knowledge portal. Note: Dashed lines – protective effect (_Beta/OR_▼); solid lines – risk effect (_Beta/OR_▲); related phenotypes are linked together. CCS–Causative Classification System, AF – arterial fibrillation.

Additionally, it is worth noting the substantial role played by rs4644832 *SERF2*, rs11666524, and rs346157 *C19orf53* in regulating the alternative splicing of the *C19orf53* gene across various tissues such as blood vessels, brain, blood cells, and heart. This regulatory mechanism contributes to increasing the diversity of the C19orf53 protein and phenotypic traits. Moreover, it may also serve as a factor for the tissue-specific regulation of protein expression levels [73].In Figure 3, we summarized the associations of the tag SNPs of the genes *C9orf16* (*BBLN*), *C11orf58*, *SERBP1*, *SERF2*, and *C19orf53* with various cardiovascular phenotypes based on the bioinformatic analysis performed here and previously [16], 17], 19].

Figure 3 reflects the substantial influence of the Hero tag SNPs on cardiovascular abnormalities. In particular, many polymorphisms affect lipid metabolism and blood pressure. *SERF2* and *C9orf16* (*BBLN*) have been almost exclusively related to changes in blood pressure and lipid metabolic abnormalities. Interestingly, *SERF2* rs4644823 G/A turned out to increase the risk of dyslipidemia while preventing hypertension and peripheral artery disease in smokers.

We revealed that all studied tag SNPs significantly influence binding with transcription factors as well. Notably, related transcription factors are mutually involved in a spectrum of overrepresented biological processes, controlling some crucial pathways of CVDs pathogenesis such as vasculogenesis, cellular response to reactive oxygen species, response to hypoxia, regulation of inflammatory response and cytokine production, as well as apoptosis (Figure 2).

Taken together with our previous research, our data provides bioinformatic evidence that Hero-proteins may be a novel link in the pathogenesis of cardiovascular pathologies like coronary artery disease and peripheral artery disease.

## 5 Conclusions

Due to the high regulatory potential of tag SNPs, genes encoding the Hero-proteins may be considered promising candidates to further study their role in CVDs. Moreover, even though in this work we presented only the genetic data, the analysis also gives a clue to further address the cardiovascular roles of the encoded proteins. Summarizing our data and the immense role of chaperones in orchestrating cellular biology, we believe that therapeutic interventions targeting the Hero-proteins may be utterly effective in fighting cardiovascular pathologies.

## Supplementary Material

Supplementary Material Details
