# Supplementary material for: Bioinformatic analysis of the regulatory potential of tagging SNPs provides evidence of the involvement of genes encoding the heat-resistant obscure (Hero) proteins in the pathogenesis of cardiovascular diseases
Source: J Integr Bioinform. 2025 Jun 3;22(1):20240043. doi: 10.1515/jib-2024-0043 (PMC12327200; doi:10.1515/jib-2024-0043)
Supplement: Supplementary file 1 — Supplementary Material Details [file j_jib-2024-0043_suppl_001.docx]

| 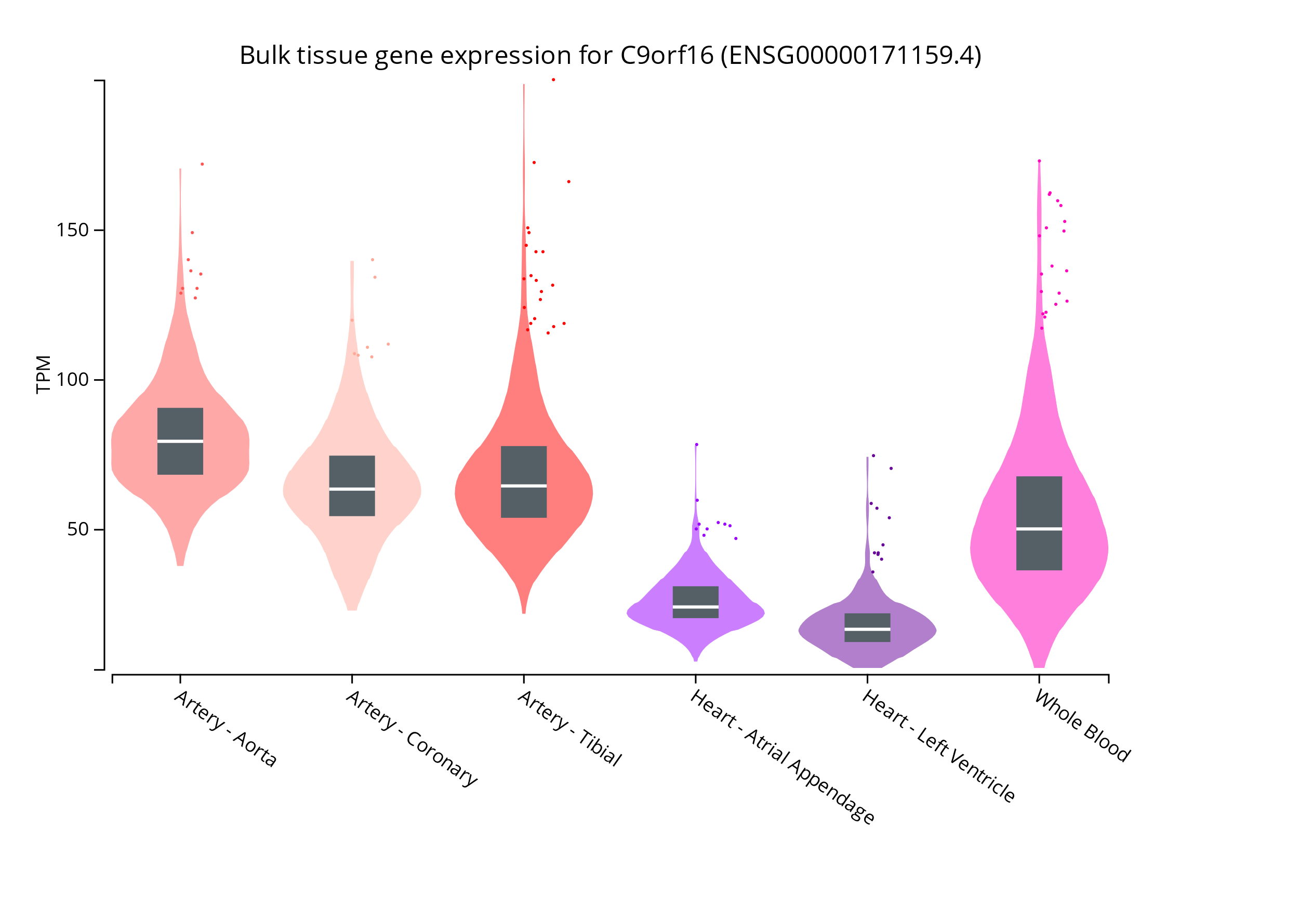  A | 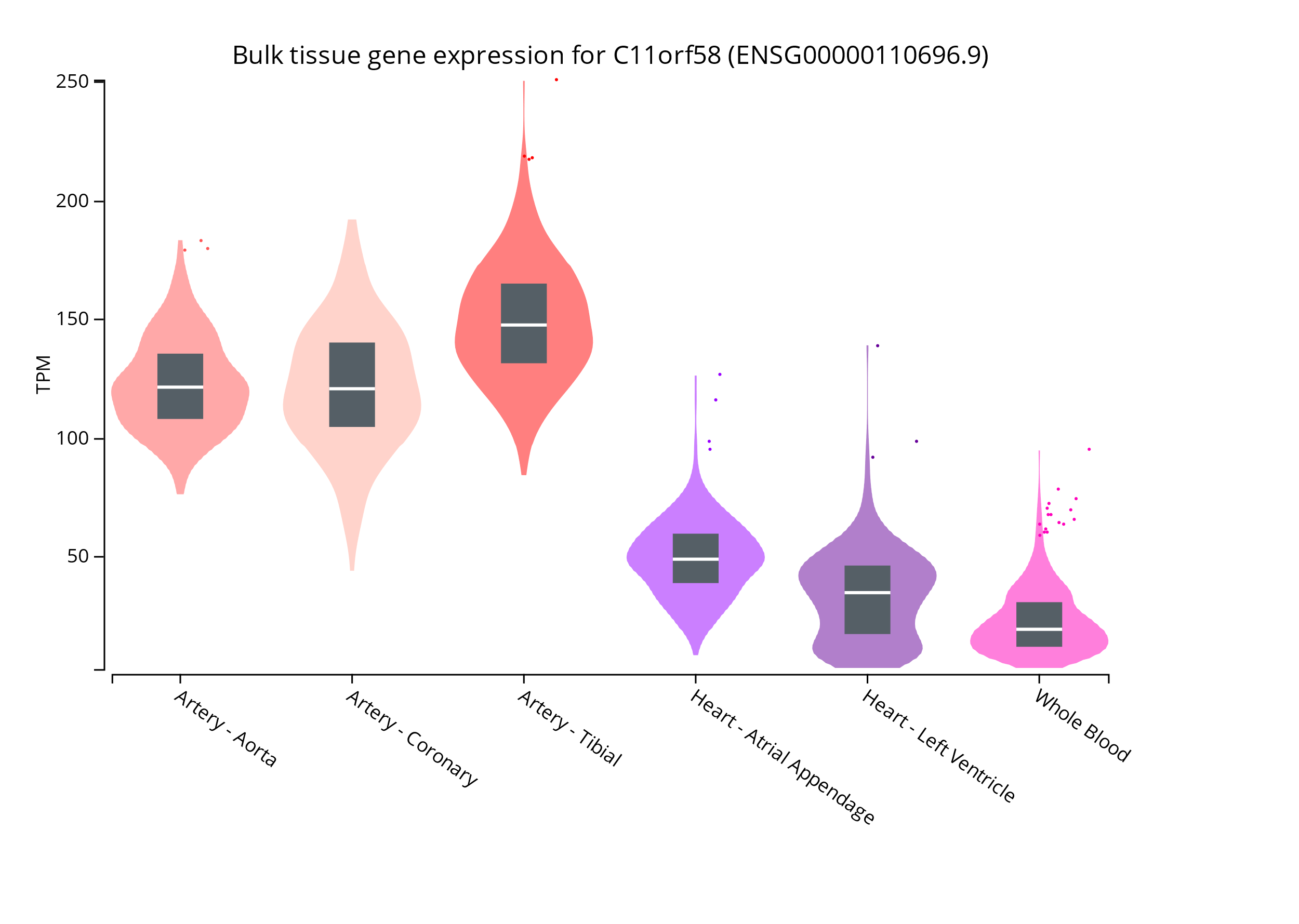  B |
| --- | --- |
| 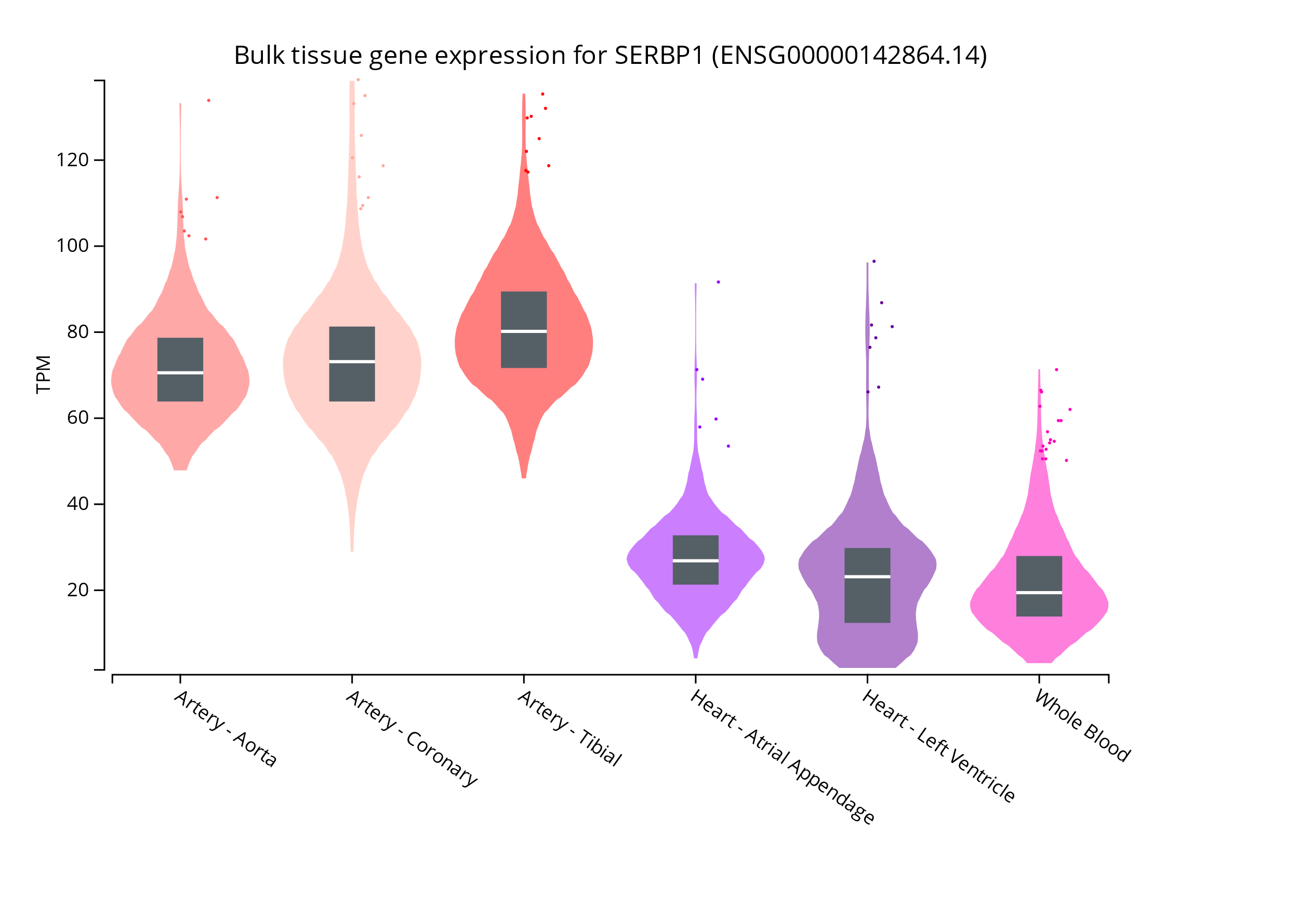  C | 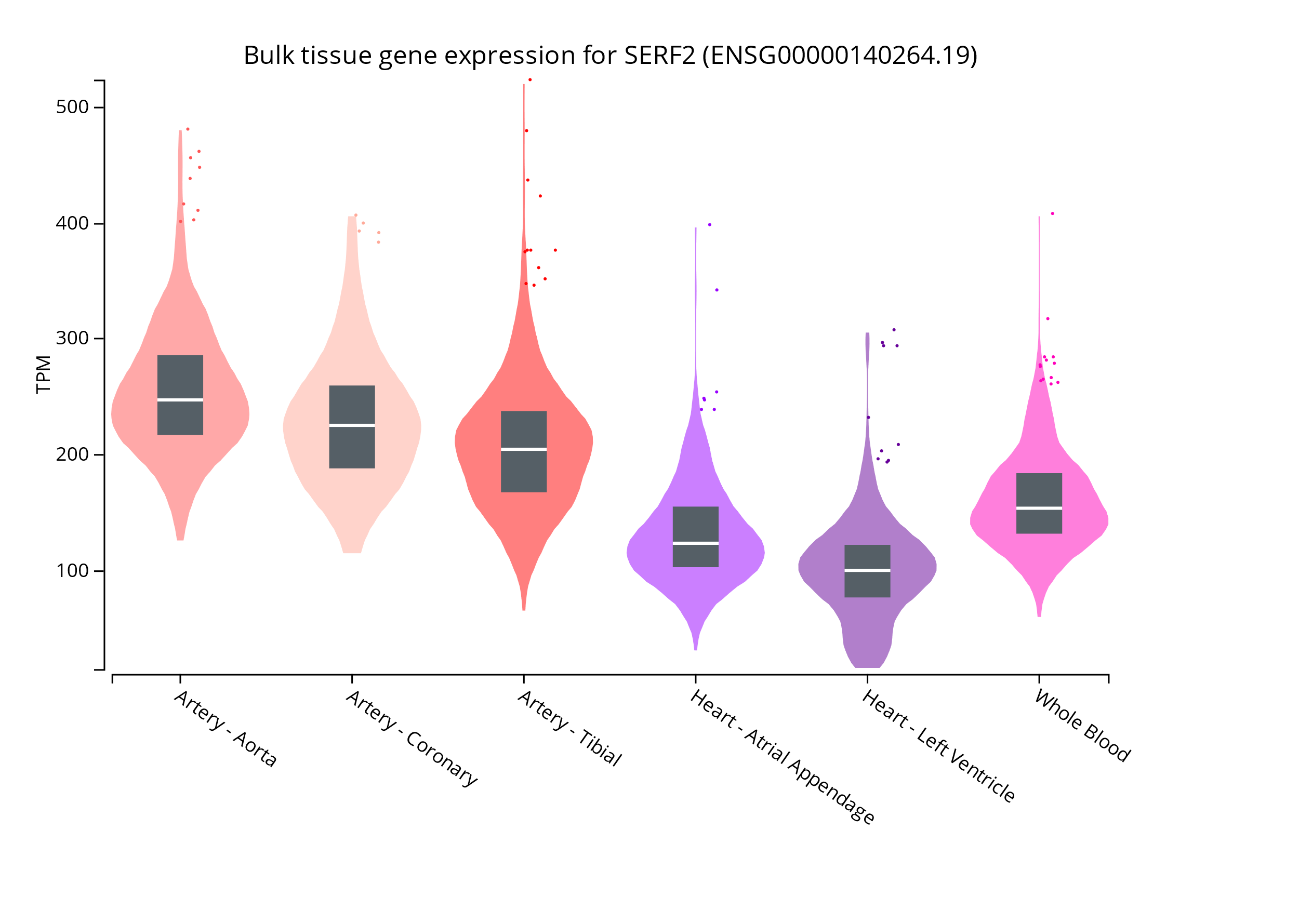  D |
| 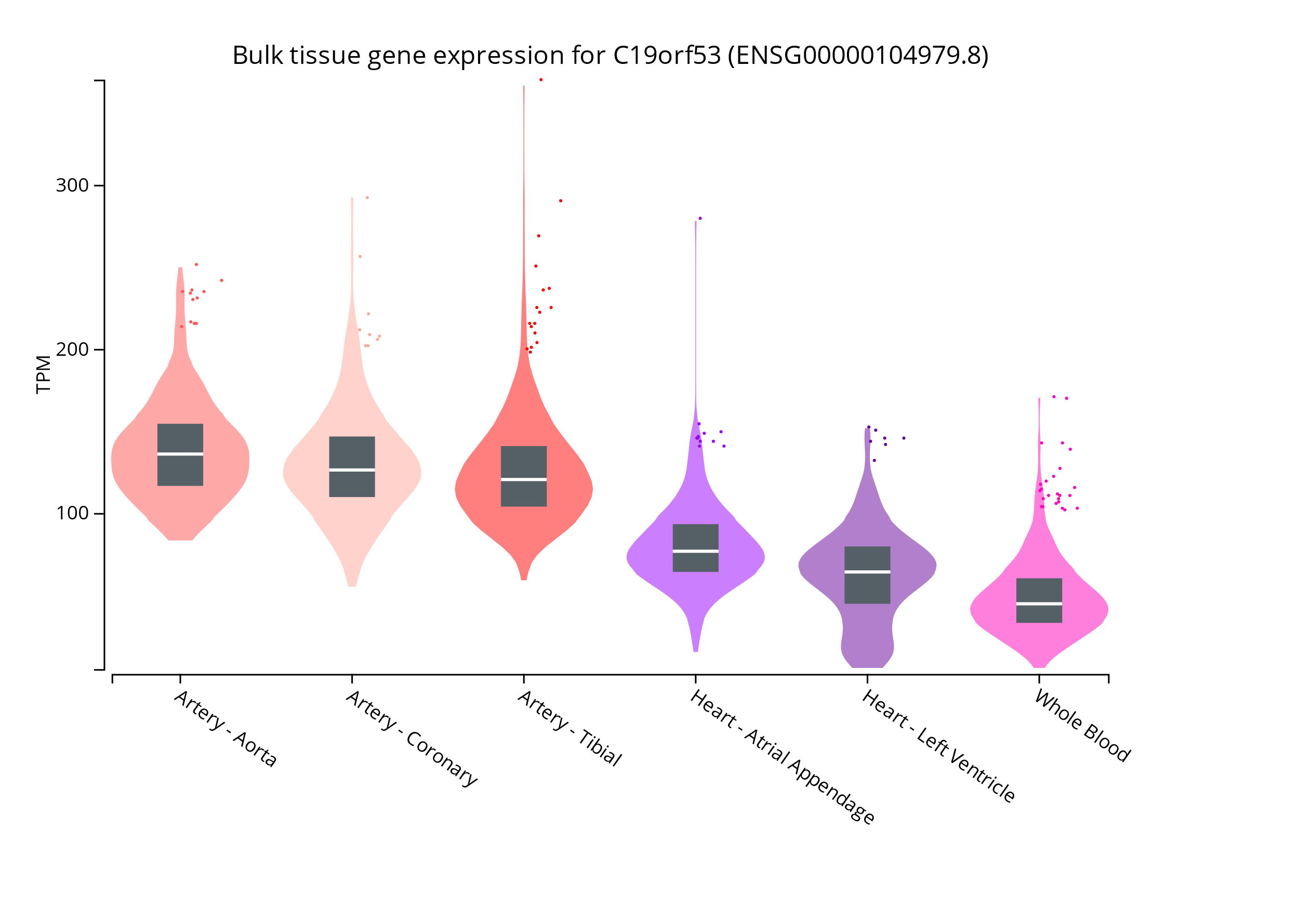  E | 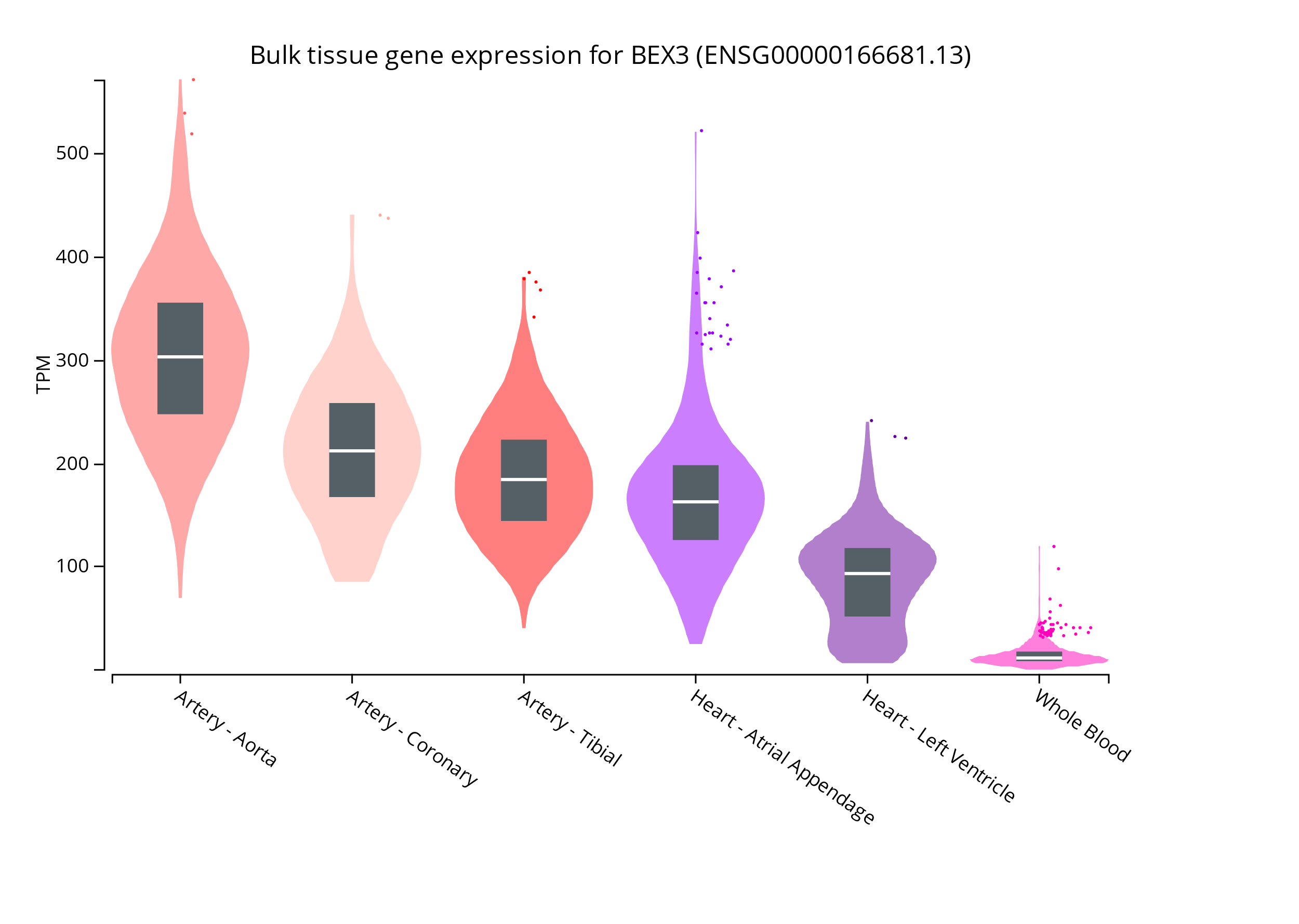  F |

Supplementary Figure 1: Expression level of genes *C9orf16* (A), *C11orf58* (B), *SERBP1* (C), *SERF2* (D), *C19orf53* (E), *BEX3* (F) in blood vessels, heart tissue, brain, whole blood (data https://gtexportal.org)

Supplementary Table 1: Preliminary analysis of regulatory potential of tagSNPs in genes encoding Hero proteins.

| № | Gene | Tag SNP | Location | RP | HaploReg | rSNPBase | Regulome |
| --- | --- | --- | --- | --- | --- | --- | --- |
| 1. | ***C9orf16*** | rs2900262 | Intron | NA | Enh 15 tis; DNAse 7 tis; 4 motifs | Dreg, RNA BPMReg, miRNAreg | 1b |
| 2. | ***C11orf58*** | rs11024032 | Intron | NA | DNAse 1 tis | Preg, Dreg, RNA BPMReg | 5 |
| 3. | ***C11orf58*** | rs3802963 | Intron | 0.0 | Prom 24 tis; DNAse 31 tis; 1 Pb | Preg, Dreg, RNA BPMReg | 4 |
| 4. | ***C11orf58*** | rs7951676 | Intron | 0.095 | 4 motifs | Preg, Dreg, RNA BPMReg | 6 |
| 5. | ***C11orf58*** | rs6677 | 3' UTR | 0.0 | Enh 2 tis; DNAse 1 tis; 1 motif | Preg, Dreg, RNA BPMReg | 5 |
| 6. | ***SERBP1*** | rs12561767 | Intron | NA | 3 motifs | Preg, RNA BPMReg | 1f |
| 7. | ***SERBP1*** | rs12566098 | Intron | NA | Enh 8 tis; DNAse 1 tis; 1 Pb; 3 motifs | Preg, Dreg, RNA BPMReg | 4 |
| 8. | ***SERF2*** | rs4644832 | Intron | 0.403 | Enh 1 tis; DNAse 1 tis | Preg, Dreg, RNA BPMReg | 4 |
| 9. | ***C19orf53*** | rs11666524 | Intron | 0.0 | Prom 24 tis; DNAse 44 tis: 7 Pb; 1 motif | Preg, Dreg, RNA BPMReg | 4 |
| 10. | ***C19orf53*** | rs2901077 | Intron | 0.0 | Enh 13 tis; DNAse 1 tis; 1 motif | Preg, Dreg, RNA BPMReg | 5 |
| 11. | ***C19orf53*** | rs346157 | Intron | NA | Enh 3 tis; DNAse 3 tis | Preg, Dreg, RNA BPMReg | 4 |
| Note:  RP – Regulatory Potential Score;  Enh – enhancer, Prom – promoter, DNAse – region of hypersensitivity to DNase-1; PB – binding site for regulatory proteins; motifs - variable regulatory DNA motifs; tis – number of tissues and organs;  Preg – proximal transcriptional regulation, Dreg – distal transcriptional regulation, RNA BPMReg – regulation mediated by RNA-binding proteins, miRNAreg – microRNA-mediated regulation;  RegulomeDB: regulatory coefficients are presented - 1b (eQTL + TF binding + any motif + DNase Footprint + DNase peak), 1d (eQTL + TF binding + any motif + DNase peak), 1f (eQTL + TF binding / DNase peak), 4 (TF binding + DNase peak), 5 (TF binding or DNase peak), 6 (Motif hit);  atSNP: the total number of DNA binding sites with TFs in the SNP region is presented (TFs were included only if the SNP had a high and very high degree of influence on the interaction of TFs with DNA)  Minor allele frequencies are presented according to 1000 Genomes Project Phase 3 allele frequencies | | | | | | | |

Supplementary Table 2: Established associations of the studied SNPs with loci of quantitative splicing traits.

| SNP Id | Gene Symbol | Variant Id | Phenotype Id | Intron Id | P-Value | NES | Tissue |
| --- | --- | --- | --- | --- | --- | --- | --- |
| rs4644832  ***SERF2***  G/A | *AC011330.5* | chr15_43792507_G_A_b38 | chr15:43632363:43632717:clu_17822:ENSG00000249839.1 | 43632363:43632717:clu_17822 | 2.0e-11 | -0.64 | Artery - Aorta |
|  | *AC011330.5* | chr15_43792507_G_A_b38 | chr15:43632363:43632717:clu_18454:ENSG00000249839.1 | 43632363:43632717:clu_18454 | 9.8e-14 | -0.54 | Artery - Tibial |
|  | *CATSPER2* | chr15_43792507_G_A_b38 | chr15:43632363:43632717:clu_17822:ENSG00000166762.16 | 43632363:43632717:clu_17822 | 2.0e-11 | -0.64 | Artery - Aorta |
|  | *CATSPER2* | chr15_43792507_G_A_b38 | chr15:43632363:43635360:clu_16669:ENSG00000166762.16 | 43632363:43635360:clu_16669 | 2.4e-7 | 0.66 | Artery - Coronary |
|  | *CATSPER2* | chr15_43792507_G_A_b38 | chr15:43632363:43632717:clu_18454:ENSG00000166762.16 | 43632363:43632717:clu_18454 | 9.8e-14 | -0.54 | Artery - Tibial |
|  | *CATSPER2* | chr15_43792507_G_A_b38 | chr15:43632363:43632717:clu_17605:ENSG00000166762.16 | 43632363:43632717:clu_17605 | 2.6e-7 | -0.54 | Heart - Atrial Appendage |
| rs11666524  ***C19orf53***  G/A | *C19orf53* | chr19_13776364_G_A_b38 | chr19:13774574:13774652:clu_19657:ENSG00000104979.8 | 13774574:13774652:clu_19657 | 2.8e-8 | 0.40 | Heart - Left Ventricle |
| rs346157  ***C19orf53***  A/G | *C19orf53* | chr19_13777478_A_G_b38 | chr19:13774574:13774652:clu_19657:ENSG00000104979.8 | 13774574:13774652:clu_19657 | 0.0000014 | 0.32 | Heart - Left Ventricle |

Supplementary Table 3: Established associations of the studied SNPs with a cis-mQTL-mediated effect on the level of methylation of CpG sites (according to the QTL base).

| Trait | Effective  Allele | Tissue | Effect Size (beta) | FDR |
| --- | --- | --- | --- | --- |
| rs2900262 ***C9orf16*** | | | | |
| cg09976142 (chr9:130955436) | NA | Blood-Monocytes CD14+ | 0.853 | 2.4×10^-5^ |
| cg09976142 (chr9:130955436) | NA | Blood | -0.592 | 0.0003 |
| cg09976142 (chr9:130955436) | NA | Blood | -0.546 | 0.001 |
| cg09976142 (chr9:130955436) | NA | Blood-T cell CD4+ naive | 0.802 | 0.002 |
| cg09976142 (chr9:130955436) | NA | Blood | -0.504 | 0.01 |
| cg09976142 (chr9:130955436) | NA | Blood | -0.543 | 0.03 |
| cg10071929 (chr9:130955135) | NA | Blood-Monocytes CD14+ | 0.732 | 0.0007 |
| cg10071929 (chr9:130955135) | NA | Blood-T cell CD4+ naive | 0.734 | 0.004 |
| cg11884704 (chr9:130854313) | NA | Blood-Monocytes CD14+ | -0.544 | 0.03 |
| cg13518265 (chr9:130910662) | NA | Blood-Monocytes CD14+ | -0.930 | 4.7×10^-5^ |
| cg13588599 (chr9:131102140) | NA | Blood-Monocytes CD14+ | 0.669 | 0.005 |
| cg13642260 (chr9:130955380) | NA | Blood-Monocytes CD14+ | 0.707 | 0.001 |
| cg13642260 (chr9:130955380) | NA | Blood-T cell CD4+ naive | 0.695 | 0.008 |
| cg14140152 (chr9:130953543) | NA | Blood-T cell CD4+ naive | -0.781 | 0.01 |
| cg24392274 (chr9:130921842) | NA | Blood-T cell CD4+ naive | 0.736 | 0.02 |
| cg24392274 (chr9:130921842) | NA | Blood | -0.455 | 0.02 |
| rs11024032 ***C11orf58*** | | | | |
| - | - | - | - | - |
| rs3802963 ***C11orf58*** | | | | |
| cg15378786 (chr11:17036137) | NA | Blood-Monocytes CD14+ | -0.564 | 8.2×10^-5^ |
| cg01977079 (chr11:17036644) | NA | Blood-T cell CD4+ naive | 0.464 | 0.03 |
| cg15378786 (chr11:17036137) | NA | Blood-T cell CD4+ naive | -0.451 | 0.03 |
| cg17284609 (chr11:16424673) | NA | Blood | 0.310 | 0.0002 |
| cg17284609 (chr11:16424673) | NA | Blood-T cell CD4+ naive | -0.523 | 0.009 |
| cg17284609 (chr11:16424673) | NA | Blood | 0.287 | 0.03 |
| rs7951676 ***C11orf58*** | | | | |
| cg18749349 (chr11:16431523) | NA | Blood | 0.243 | 0.006 |
| rs6677 ***C11orf58*** | | | | |
| - | - | - | - | - |
| rs12561767 ***SERBP1*** | | | | |
| cg08660285 (chr1:67390436) | NA | Blood-Monocytes CD14+ | -0.428 | 0.0002 |
| cg24364144 (chr1:67875067) | NA | Blood | -0.230 | 0.02 |
| cg24364144 (chr1:67875067) | NA | Blood-T cell CD4+ naive | 0.452 | 0.03 |
| rs12566098 ***SERBP1*** | | | | |
| cg08660285 (chr1:67390436) | NA | Blood-Monocytes CD14+ | -0.329 | 0.01 |
| cg24364144 (chr1:67875067) | NA | Blood | -0.246 | 0.02 |
| cg24364144 (chr1:67875067) | NA | Blood-T cell CD4+ naive | 0.486 | 0.03 |
| rs4644832 ***SERF2*** | | | | |
| cg12861797 (chr15:43585817) | NA | Blood | 0.418 | 2.7×10^-8^ |
| cg12861797 (chr15:43585817) | NA | Blood | 0.364 | 5.9×10^-7^ |
| cg06158227 (chr15:43662311) | NA | Blood-T cell CD4+ naive | -0.427 | 0.02 |
| cg12032620 (chr15:43802735) | NA | Blood-T cell CD4+ naive | -0.404 | 0.04 |
| cg12861797 (chr15:43585817) | NA | Blood | 0.405 | 8.5×10^-7^ |
| cg12861797 (chr15:43585817) | NA | Blood | 0.327 | 1.5×10^-5^ |
| cg12861797 (chr15:43585817) | NA | Blood-Monocytes CD14+ | -0.479 | 0.0005 |
| cg12861797 (chr15:43585817) | NA | Blood | 0.314 | 0.001 |
| cg12861797 (chr15:43585817) | NA | Blood-T cell CD4+ naive | -0.432 | 0.01 |
| cg16445139 (chr15:43663125) | NA | Blood-Monocytes CD14+ | 0.429 | 0.005 |
| cg16487861 (chr15:43786615) | NA | Blood-Monocytes CD14+ | 0.564 | 0.0008 |
| cg21033855 (chr15:43662581) | NA | Blood | -0.260 | 0.01 |
| cg21245717 (chr15:43585934) | NA | Blood-Monocytes CD14+ | -0.536 | 0.0001 |
| cg21245717 (chr15:43585934) | NA | Blood-T cell CD4+ naive | -0.467 | 0.01 |
| rs11666524 ***C19orf53*** | | | | |
| cg09254823 (chr19:13885429) | NA | Blood-Monocytes CD14+ | -0.617 | 6.8×10^-6^ |
| cg09254823 (chr19:13885429) | NA | Blood-T cell CD4+ naive | -0.521 | 0.02 |
| cg09952620 (chr19:13885098) | NA | Blood | -0.247 | 0.0004 |
| cg09952620 (chr19:13885098) | NA | Blood-Monocytes CD14+ | -0.463 | 0.006 |
| cg13587756 (chr19:13889434) | NA | Blood-Monocytes CD14+ | 0.510 | 0.001 |
| cg21192260 (chr19:13884760) | NA | Blood | -0.338 | 1.5×10^-6^ |
| cg21192260 (chr19:13884760) | NA | Blood-Monocytes CD14+ | -0.632 | 3.0×10^-6^ |
| cg21192260 (chr19:13884760) | NA | Blood | -0.272 | 0.006 |
| rs2901077 ***C19orf53*** | | | | |
| cg25722029 (chr19:13907696) | NA | Blood-Monocytes CD14+ | -0.764 | 0.0004 |
| cg25722029 (chr19:13907696) | NA | Blood-T cell CD4+ naive | -0.824 | 0.004 |
| rs346157 ***C19orf53*** | | | | |
| cg16474696 (chr19:13875014) | NA | Blood | 0.526 | 7.1×10^-22^ |
| cg01530988 (chr19:13875289) | NA | Blood-Monocytes CD14+ | 0.387 | 0.008 |
| cg16474696 (chr19:13875014) | NA | Blood | 0.487 | 1.9×10^-17^ |
| cg16474696 (chr19:13875014) | NA | Blood | 0.360 | 1.2×10^-8^ |
| cg16474696 (chr19:13875014) | NA | Blood | 0.348 | 1.9×10^-8^ |
| cg16474696 (chr19:13875014) | NA | Blood-Monocytes CD14+ | 0.398 | 0.002 |
| cg16474696 (chr19:13875014) | NA | Blood-T cell CD4+ naive | 0.429 | 0.02 |
| cg21192260 (chr19:13884760) | NA | Blood-Monocytes CD14+ | -0.495 | 3.0×10^-5^ |
| cg25755428 (chr19:13875111) | NA | Blood | 0.506 | 1.3×10^-19^ |
| cg25755428 (chr19:13875111) | NA | Blood | 0.431 | 9.6×10^-13^ |
| cg25755428 (chr19:13875111) | NA | Blood | 0.332 | 1.7×10^-7^ |
| cg25755428 (chr19:13875111) | NA | Blood | 0.333 | 4.5×10^-7^ |
| cg25755428 (chr19:13875111) | NA | Blood-Monocytes CD14+ | 0.437 | 0.0004 |
| cg25755428 (chr19:13875111) | NA | Blood | 0.259 | 0.001 |
| cg25755428 (chr19:13875111) | NA | Blood-T cell CD4+ naive | 0.459 | 0.009 |

Supplementary Table 4: Proven effects of tag SNPs on tissue-specific histone modifications.

| SNP | Histone marks | 1 | 2 | 3 | 4 | 5 |
| --- | --- | --- | --- | --- | --- | --- |
| *C9orf16* rs2900262 (T/**C**) | H3K4me1 | E | - | E | E | E |
|  | H3K4me3 | P | - | - | - | - |
|  | H3K27ac | E | - | E | E | E |
|  | H3K9ac | P | - | - | - | - |
| *C11orf58* rs11024032 (C/**T**) | H3K4me1 | E | - | E | - | - |
|  | H3K4me3 | - | - | - | - | - |
|  | H3K27ac | E | - | - | - | - |
|  | H3K9ac | - | - | - | - | - |
|  | DNase | 🗸 | - | - | - | - |
| *C11orf58* rs3802963 (C/**G**) | H3K4me1 | E | - | E | - | - |
|  | H3K4me3 | P | P | P | P | P |
|  | H3K27ac | E | E | E | E | - |
|  | H3K9ac | P | - | - | - | - |
| *C11orf58* rs7951676 (G/**T**) | H3K4me1 | E | - | - | - | - |
|  | H3K4me3 | P | - | - | - | - |
|  | H3K27ac | E | - | - | - | - |
|  | H3K9ac | P | - | - | - | - |
| *C11orf58* rs6677 (T/**G**) | H3K27ac | E | - | - | - | - |
| *SERBP1* rs12561767 (G/**A**) | H3K4me1 | E | - | E | E | E |
|  | H3K27ac | E | - | - | - | E |
| *SERBP1* rs12566098 (C/**G**) | H3K4me1 | E | - | - | - | E |
|  | H3K4me3 | P | - | - | - | - |
|  | H3K27ac | E | - | - | - | E |
|  | H3K9ac | P | - | - | - | - |
| *SERF2* rs4644832 (G/**A**) | H3K4me1 | E | - | - | - | - |
|  | H3K4me3 | P | P | P | P | P |
|  | H3K27ac | E | P | P | P | P |
|  | H3K9ac | P | - | - | - | - |
|  | DNase | 🗸 | - | - | - | - |
| *C19orf53* rs11666524 (G/**A**) | H3K4me1 | E | E | E | E | E |
|  | H3K4me3 | P | - | - | - | P |
|  | H3K27ac | E | - | - | - | E |
|  | H3K9ac | P | - | - | - | - |
| *C19orf53* rs2901077 (C/**T**) | H3K4me1 | E | - | - | - | E |
|  | H3K4me3 | P | - | - | - | - |
|  | H3K27ac | E | - | - | - | E |
|  | H3K9ac | P | - | - | - | - |
| *C19orf53* rs346157 (A/**G**) | H3K4me1 | E | - | - | - | E |
|  | H3K4me3 | P | - | - | - | - |
|  | H3K27ac | E | - | - | - | E |
|  | H3K9ac | P | - | - | - | - |
| The Table presents the data of the bioinformatic resource *HaploReg v4.1.*;  H3K4me1—histone H3 lysine 4 mo--methylation; H3K4me3—histone H3 lysine 4 tri-methylation; H3K9ac—the acetylation at the 9th lysine residues of the histone H3 protein; H3K27ac—acetylation of lysine 27 on histone H3 protein subunit; effect alleles are marked in bold. E—histone modification in the enhancer region; P—histone modification at the promoter region. 1—Cells from peripheral blood (any); 2 – aorta; 3 - right atrium; 4 – left ventricle; 5 - right ventricle; Effect alleles are marked in bold. | | | | | | |

Supplementary Table 5: Analysis of the effect of rs11024032 *C11orf58* on the binding of DNA to transcription factors.

| № | Ref/  SNP allele^1^ | TF^2^ | GAIN  /LOSS^3^ | Motif^4^ | P-Value SNP impact^5^ | P-Value Ref^6^ | P-Value SNP^7^ |
| --- | --- | --- | --- | --- | --- | --- | --- |
| 1 | C/T | TEAD1 | gain | MA0090.1 | 0.0001 | 0.158 | 0.012 |
| 2 | C/T | NR1H4 | gain | NR1H4_1 | 0.0002 | 0.082 | 0.008 |
| 3 | C/T | FOXH1 | gain | MA0479.1 | 0.002 | 0.113 | 0.005 |
| 4 | C/T | POU2F1 | gain | POU2F1_2 | 0.002 | 0.112 | 0.002 |
| 5 | C/T | CEBP | gain | CEBP_1 | 0.002 | 0.739 | 0.017 |
| 6 | C/T | NR1H4 | gain | NR1H4_2 | 0.003 | 0.140 | 0.020 |
| 7 | C/T | CCDC6 | gain | CCDC6_1 | 0.004 | 0.272 | 0.012 |
| 8 | C/T | POU1F1 | gain | POU1F1_2 | 0.005 | 0.421 | 0.024 |
| 9 | C/T | TEAD1 | gain | TEAD1_2 | 0.005 | 0.118 | 0.010 |
| 10 | C/T | SIX1 | gain | SIX1_1 | 0.006 | 0.291 | 0.026 |
| 11 | C/T | CRX | gain | CRX_2 | 0.007 | 0.177 | 0.011 |
| 12 | C/T | GATA | gain | GATA_1 | 0.007 | 0.557 | 0.032 |
| 13 | C/T | OBOX6 | gain | OBOX6_1 | 0.007 | 0.076 | 0.006 |
| 14 | C/T | POU2F2 | gain | POU2F2_4 | 0.008 | 0.310 | 0.036 |
| 15 | C/T | NFAT5 | gain | NFAT5_1 | 0.008 | 0.213 | 0.018 |
| 16 | C/T | NFKB | gain | NFKB_disc1 | 0.009 | 0.052 | 0.007 |
| 17 | C/T | RELA | gain | RELA_2 | 0.011 | 0.091 | 0.008 |
| 18 | C/T | RUNX1 | gain | RUNX1_5 | 0.012 | 0.220 | 0.029 |
| 19 | C/T | AP1 | gain | AP1_disc1 | 0.015 | 0.436 | 0.048 |
| 20 | C/T | TEAD4 | gain | TEAD4_1 | 0.024 | 0.210 | 0.021 |
| 21 | C/T | GATA1 | gain | GATA1_2 | 0.028 | 0.640 | 0.036 |
| 22 | C/T | HMGA1 | gain | HMGA1_1 | 0.029 | 0.108 | 0.006 |
| 23 | C/T | GSC2 | gain | GSC2_1 | 0.031 | 0.396 | 0.050 |
| 24 | C/T | SOX10 | gain | SOX10_3 | 0.035 | 0.150 | 0.031 |
| 25 | C/T | EP300 | gain | EP300_disc10 | 0.040 | 0.169 | 0.037 |
| 26 | C/T | IRF7 | gain | IRF7_3 | 0.049 | 0.160 | 0.038 |
| 27 | C/T | AR | loss | AR_6 | 0.00004 | 0.00005 | 0.118 |
| 28 | C/T | NFAT | loss | NFAT_1 | 0.0001 | 0.011 | 0.259 |
| 29 | C/T | AR | loss | MA0007.2 | 0.001 | 0.004 | 0.154 |
| 30 | C/T | NR3C1 | loss | MA0113.2 | 0.001 | 0.004 | 0.083 |
| 31 | C/T | PGR | loss | PGR_2 | 0.001 | 0.023 | 0.328 |
| 32 | C/T | EP300 | loss | EP300_disc4 | 0.002 | 0.008 | 0.136 |
| 33 | C/T | AR | loss | AR_2 | 0.003 | 0.005 | 0.178 |
| 34 | C/T | IRF1 | loss | IRF1_3 | 0.005 | 0.008 | 0.143 |
| 35 | C/T | EP300 | loss | EP300_disc5 | 0.007 | 0.037 | 0.534 |
| 36 | C/T | BATF | loss | BATF_disc2 | 0.008 | 0.015 | 0.134 |
| 37 | C/T | AR | loss | AR_8 | 0.011 | 0.012 | 0.112 |
| 38 | C/T | BACH2 | loss | BACH2_1 | 0.011 | 0.041 | 0.776 |
| 39 | C/T | STAT | loss | STAT_disc3 | 0.012 | 0.039 | 0.279 |
| 40 | C/T | Spz1 | loss | MA0111.1 | 0.012 | 0.022 | 0.216 |
| 41 | C/T | NR3C1 | loss | NR3C1_5 | 0.013 | 0.025 | 0.446 |
| 42 | C/T | AR | loss | AR_7 | 0.014 | 0.009 | 0.072 |
| 43 | C/T | STAT2::STAT1 | loss | MA0517.1 | 0.017 | 0.022 | 0.183 |
| 44 | C/T | Spi1 | loss | MA0080.3 | 0.020 | 0.038 | 0.318 |
| 45 | C/T | HINFP | loss | MA0131.1 | 0.020 | 0.041 | 0.407 |
| 46 | C/T | SPI1 | loss | SPI1_3 | 0.023 | 0.024 | 0.190 |
| 47 | C/T | NANOG | loss | NANOG_disc3 | 0.027 | 0.012 | 0.102 |
| 48 | C/T | CACD | loss | CACD_1 | 0.032 | 0.038 | 0.231 |
| 49 | C/T | AR | loss | AR_3 | 0.042 | 0.019 | 0.111 |
| 50 | C/T | SPI1 | loss | SPI1_1 | 0.043 | 0.035 | 0.238 |
|  | T^8^ | positive regulation of leukocyte adhesion to vascular endothelial cell (GO:1904996; FDR=**0.046**);  positive regulation of non-canonical NF-kappaB signal transduction (GO:1901224; FDR=**0.012**) | | | | | |
|  | C^9^ | - | | | | | |
| 1 – reference (Ref) / alternative (SNP) allele;  2 – TF - transcription factor;  3 – binding of TF to the reference (LOSS) / alternative (GAIN) allele;  4 – binding sites with high affinity for TF;  5 – p value statistically confirming the potential gain or loss of function of the genomic region with SNP in terms of transcription factor binding;  6 – p-value for assessing the binding of TF to the Ref allele;  7 – p-value for assessing the binding of TF to the SNP allele;  8 – biological processes pathogenetically significant for IS, in which TFs that bind to the alternative allele are jointly involved (data from the Gene Ontology resource; http://geneontology.org/);  9 – biological processes pathogenetically significant for AI, in which TFs that bind to the reference allele are jointly involved (data from the Gene Ontology resource; http://geneontology.org/) | | | | | | | |

Supplementary Table 6: Analysis of the effect of rs3802963 *C11orf58* on the binding of DNA to transcription factors.

| № | Ref/SNP allele ^1^ | TF^2^ | GAIN  /LOSS^3^ | Motif^4^ | P-Value SNP impact^5^ | P-Value Ref^6^ | P-Value SNP^7^ |
| --- | --- | --- | --- | --- | --- | --- | --- |
| 1 | C/G | PAX4 | gain | PAX4_1 | 0.0000005 | 0.167 | 0.00001 |
| 2 | C/G | SPDEF | gain | SPDEF_3 | 0.000 | 0.239 | 0.008 |
| 3 | C/G | SEF1 | gain | SEF1_1 | 0.003 | 0.138 | 0.020 |
| 4 | C/G | SOX21 | gain | SOX21_3 | 0.006 | 0.359 | 0.017 |
| 5 | C/G | ZBTB6 | gain | ZBTB6_1 | 0.011 | 0.587 | 0.036 |
| 6 | C/G | E2F1 | gain | E2F1_14 | 0.011 | 0.091 | 0.007 |
| 7 | C/G | HNF4 | gain | HNF4_disc3 | 0.015 | 0.119 | 0.015 |
| 8 | C/G | SIN3A | gain | SIN3A_disc1 | 0.016 | 0.120 | 0.013 |
| 9 | C/G | E2F1 | gain | E2F1_18 | 0.017 | 0.088 | 0.007 |
| 10 | C/G | E2F1 | gain | E2F1_15 | 0.020 | 0.219 | 0.017 |
| 11 | C/G | SOX10 | gain | SOX10_5 | 0.025 | 0.167 | 0.036 |
| 12 | C/G | RFX1 | gain | RFX1_3 | 0.032 | 0.094 | 0.015 |
| 13 | C/G | HNF4A | loss | HNF4A_5 | 0 | 0.00003 | 0.223 |
| 14 | C/G | EGR1 | loss | EGR1_disc2 | 0.0005 | 0.008 | 0.194 |
| 15 | C/G | SRY | loss | SRY_6 | 0.002 | 0.019 | 0.203 |
| 16 | C/G | ZIC3 | loss | ZIC3_2 | 0.004 | 0.026 | 0.180 |
| 17 | C/G | ELF1 | loss | ELF1_disc1 | 0.004 | 0.003 | 0.080 |
| 18 | C/G | ZIC1 | loss | ZIC1_2 | 0.004 | 0.030 | 0.182 |
| 19 | C/G | ZIC3 | loss | ZIC3_4 | 0.005 | 0.016 | 0.211 |
| 20 | C/G | ZIC2 | loss | ZIC2_2 | 0.007 | 0.034 | 0.188 |
| 21 | C/G | ETS | loss | ETS_disc2 | 0.009 | 0.006 | 0.144 |
| 22 | C/G | NFE2L2 | loss | NFE2L2_1 | 0.010 | 0.006 | 0.108 |
| 23 | C/G | ELK4 | loss | MA0076.2 | 0.010 | 0.011 | 0.096 |
| 24 | C/G | SPIC | loss | SPIC_2 | 0.017 | 0.049 | 0.328 |
| 25 | C/G | E2F1 | loss | E2F1_5 | 0.018 | 0.042 | 0.307 |
| 26 | C/G | FEV | loss | FEV_1 | 0.019 | 0.029 | 0.255 |
| 27 | C/G | AIRE | loss | AIRE_2 | 0.020 | 0.028 | 0.158 |
| 28 | C/G | SOX9 | loss | SOX9_4 | 0.025 | 0.034 | 0.189 |
| 29 | C/G | FLI1 | loss | MA0475.1 | 0.026 | 0.047 | 0.480 |
| 30 | C/G | ZIC4 | loss | ZIC4_1 | 0.027 | 0.027 | 0.138 |
| 31 | C/G | MYB | loss | MYB_2 | 0.027 | 0.031 | 0.213 |
| 32 | C/G | ETV6 | loss | ETV6_1 | 0.030 | 0.021 | 0.131 |
| 33 | C/G | ZSCAN4 | loss | ZSCAN4_1 | 0.030 | 0.014 | 0.074 |
| 34 | C/G | E2F4 | loss | MA0470.1 | 0.032 | 0.047 | 0.385 |
| 35 | C/G | HDAC2 | loss | HDAC2_disc4 | 0.039 | 0.025 | 0.217 |
| 36 | C/G | ELF5 | loss | ELF5_3 | 0.049 | 0.011 | 0.058 |
|  | G^8^ | regulation of apoptotic process (GO:0042981; FDR=**0.047**) | | | | | |
|  | C^9^ | regulation of transcription from RNA polymerase II promoter in response to hypoxia (GO:0061418; FDR=**0.019**) | | | | | |
| 1 – reference (Ref) / alternative (SNP) allele;  2 – TF - transcription factor;  3 – binding of TF to the reference (LOSS) / alternative (GAIN) allele;  4 – binding sites with high affinity for TF;  5 – p value statistically confirming the potential gain or loss of function of the genomic region with SNP in terms of transcription factor binding;  6 – p-value for assessing the binding of TF to the Ref allele;  7 – p-value for assessing the binding of TF to the SNP allele;  8 – biological processes pathogenetically significant for IS, in which TFs that bind to the alternative allele are jointly involved (data from the Gene Ontology resource; http://geneontology.org/);  9 – biological processes pathogenetically significant for AI, in which TFs that bind to the reference allele are jointly involved (data from the Gene Ontology resource; http://geneontology.org/) | | | | | | | |

Supplementary Table 7: Analysis of the effect of rs6677 *C11orf58* on the binding of DNA to transcription factors.

| № | Ref/SNP allele^1^ | TF^2^ | GAIN  /LOSS^3^ | Motif^4^ | P-Value SNP impact^5^ | P-Value Ref^6^ | P-Value SNP^7^ |
| --- | --- | --- | --- | --- | --- | --- | --- |
| 1 | T/G | NR1H | gain | NR1H_3 | 0.00005 | 0.115 | 0.0001 |
| 2 | T/G | HIC1 | gain | HIC1_4 | 0.001 | 0.845 | 0.028 |
| 3 | T/G | GLIS1 | gain | GLIS1_1 | 0.001 | 0.211 | 0.006 |
| 4 | T/G | RFX1 | gain | RFX1_3 | 0.002 | 0.062 | 0.004 |
| 5 | T/G | TFAP2 | gain | TFAP2_3 | 0.002 | 0.363 | 0.021 |
| 6 | T/G | RARA | gain | RARA_8 | 0.002 | 0.263 | 0.011 |
| 7 | T/G | TBX5 | gain | TBX5_1 | 0.002 | 0.446 | 0.018 |
| 8 | T/G | ZNF628 | gain | ZNF628_1 | 0.003 | 0.304 | 0.048 |
| 9 | T/G | ETV4 | gain | NR1H_3 | 0.00005 | 0.115 | 0.000 |
| 10 | T/G | RFX5 | gain | HIC1_4 | 0.001 | 0.845 | 0.028 |
| 11 | T/G | REL | gain | GLIS1_1 | 0.001 | 0.211 | 0.006 |
| 12 | T/G | TFAP2A | gain | RFX1_3 | 0.002 | 0.062 | 0.004 |
| 13 | T/G | HEY2 | gain | TFAP2_3 | 0.002 | 0.363 | 0.021 |
| 14 | T/G | ERG | gain | RARA_8 | 0.002 | 0.263 | 0.011 |
| 15 | T/G | ELF3 | gain | TBX5_1 | 0.002 | 0.446 | 0.018 |
| 16 | T/G | NFKB | gain | ZNF628_1 | 0.003 | 0.304 | 0.048 |
| 17 | T/G | SMAD3 | gain | NR1H_3 | 0.000 | 0.115 | 0.000 |
| 18 | T/G | LHX9 | gain | HIC1_4 | 0.001 | 0.845 | 0.028 |
| 19 | T/G | IRF9 | gain | GLIS1_1 | 0.001 | 0.211 | 0.006 |
| 20 | T/G | CTCF | gain | RFX1_3 | 0.002 | 0.062 | 0.004 |
| 21 | T/G | NKX2-5 | gain | TFAP2_3 | 0.002 | 0.363 | 0.021 |
| 22 | T/G | PKNOX2 | gain | RARA_8 | 0.002 | 0.263 | 0.011 |
| 23 | T/G | ETS1 | loss | ETS1_1 | 0 | 0.0000004 | 0.061 |
| 24 | T/G | ELF5 | loss | ELF5_2 | 0.00001 | 0.00004 | 0.782 |
| 25 | T/G | SPIB | loss | MA0081.1 | 0.0001 | 0.003 | 0.114 |
| 26 | T/G | EHF | loss | EHF_1 | 0.000 | 0.001 | 0.099 |
| 27 | T/G | STAT5A | loss | STAT5A_2 | 0.000 | 0.001 | 0.132 |
| 28 | T/G | ELF4 | loss | ELF4_1 | 0.000 | 0.004 | 0.906 |
| 29 | T/G | ELF3 | loss | ELF3_1 | 0.000 | 0.001 | 0.055 |
| 30 | T/G | SPI1 | loss | SPI1_1 | 0.000 | 0.005 | 0.106 |
| 31 | T/G | ELK1 | loss | ELK1_2 | 0.000 | 0.002 | 0.180 |
| 32 | T/G | SPIB | loss | SPIB_1 | 0.000 | 0.002 | 0.756 |
| 33 | T/G | ETS | loss | ETS_2 | 0.000 | 0.002 | 0.591 |
| 34 | T/G | Spi1 | loss | MA0080.3 | 0.001 | 0.017 | 0.399 |
| 35 | T/G | GABPA | loss | MA0062.2 | 0.001 | 0.002 | 0.089 |
| 36 | T/G | MYC | loss | MYC_disc6 | 0.001 | 0.012 | 0.157 |
| 37 | T/G | ETV4 | loss | ETV4_2 | 0.001 | 0.001 | 0.313 |
| 38 | T/G | ELK1 | loss | ELK1_3 | 0.001 | 0.004 | 0.934 |
| 39 | T/G | ETV6 | loss | ETV6_2 | 0.001 | 0.005 | 0.989 |
| 40 | T/G | ETS1 | loss | ETS1_4 | 0.001 | 0.001 | 0.147 |
| 41 | T/G | ETS1 | loss | ETS1_2 | 0.001 | 0.004 | 0.118 |
| 42 | T/G | SPDEF | loss | SPDEF_5 | 0.001 | 0.002 | 0.694 |
| 43 | T/G | ELK4 | loss | ELK4_1 | 0.001 | 0.003 | 0.050 |
| 44 | T/G | ETS | loss | ETS_1 | 0.002 | 0.004 | 0.249 |
| 45 | T/G | ETS1 | loss | ETS1_3 | 0.002 | 0.005 | 0.300 |
| 46 | T/G | ZNF143 | loss | ZNF143_2 | 0.002 | 0.036 | 0.520 |
| 47 | T/G | STAT6 | loss | STAT6_2 | 0.002 | 0.009 | 0.531 |
| 48 | T/G | FEV | loss | FEV_2 | 0.002 | 0.001 | 0.079 |
| 49 | T/G | ELK1 | loss | MA0028.1 | 0.002 | 0.005 | 0.178 |
| 50 | T/G | NFATC2 | loss | NFATC2_1 | 0.003 | 0.015 | 0.434 |
| 51 | T/G | ELF5 | loss | ELF5_3 | 0.003 | 0.002 | 0.071 |
| 52 | T/G | GABP | loss | GABP_1 | 0.003 | 0.029 | 0.278 |
| 53 | T/G | ITGB2 | loss | ITGB2_1 | 0.003 | 0.004 | 0.071 |
| 54 | T/G | SPDEF | loss | SPDEF_1 | 0.003 | 0.006 | 0.126 |
| 55 | T/G | STAT | loss | STAT_disc3 | 0.004 | 0.024 | 0.339 |
| 56 | T/G | IRF | loss | IRF_1 | 0.004 | 0.012 | 0.302 |
| 57 | T/G | NFAT | loss | NFAT_2 | 0.004 | 0.007 | 0.267 |
| 58 | T/G | ELF5 | loss | MA0136.1 | 0.004 | 0.010 | 0.264 |
| 59 | T/G | FOXJ3 | loss | FOXJ3_5 | 0.004 | 0.021 | 0.581 |
| 60 | T/G | ELF5 | loss | ELF5_1 | 0.004 | 0.007 | 0.329 |
| 61 | T/G | RXRA | loss | RXRA_disc4 | 0.004 | 0.035 | 0.431 |
| 62 | T/G | STAT | loss | STAT_1 | 0.004 | 0.021 | 0.227 |
| 63 | T/G | SRY | loss | SRY_1 | 0.004 | 0.006 | 0.811 |
| 64 | T/G | ELF1 | loss | MA0473.1 | 0.004 | 0.003 | 0.064 |
| 65 | T/G | SPI1 | loss | ETS1_1 | 0 | 0.0000004 | 0.061 |
| 66 | T/G | ETS | loss | ELF5_2 | 0.00001 | 0.00004 | 0.782 |
| 67 | T/G | SPI1 | loss | MA0081.1 | 0.0001 | 0.003 | 0.114 |
| 68 | T/G | HNF4 | loss | EHF_1 | 0.0002 | 0.001 | 0.099 |
| 69 | T/G | ELK3 | loss | STAT5A_2 | 0.0002 | 0.001 | 0.132 |
| 70 | T/G | BCL | loss | ELF4_1 | 0.0002 | 0.004 | 0.906 |
| 71 | T/G | STAT | loss | ELF3_1 | 0.0003 | 0.001 | 0.055 |
| 72 | T/G | ETV5 | loss | SPI1_1 | 0.0003 | 0.005 | 0.106 |
| 73 | T/G | ELK1 | loss | ELK1_2 | 0.0004 | 0.002 | 0.180 |
| 74 | T/G | NRF1 | loss | SPIB_1 | 0.0004 | 0.002 | 0.756 |
| 75 | T/G | IRF9 | loss | ETS_2 | 0.0004 | 0.002 | 0.591 |
| 76 | T/G | FOXG1 | loss | MA0080.3 | 0.001 | 0.017 | 0.399 |
| 77 | T/G | SPI1 | loss | MA0062.2 | 0.001 | 0.002 | 0.089 |
| 78 | T/G | REL | loss | MYC_disc6 | 0.001 | 0.012 | 0.157 |
| 79 | T/G | ELF3 | loss | ETV4_2 | 0.001 | 0.001 | 0.313 |
| 80 | T/G | ETS | loss | ELK1_3 | 0.001 | 0.004 | 0.934 |
| 81 | T/G | AHR::ARNT | loss | ETV6_2 | 0.001 | 0.005 | 0.989 |
| 82 | T/G | PTEN | loss | ETS1_4 | 0.001 | 0.001 | 0.147 |
| 83 | T/G | CREB3L1 | loss | ETS1_2 | 0.001 | 0.004 | 0.118 |
| 84 | T/G | NR3C1 | loss | SPDEF_5 | 0.001 | 0.002 | 0.694 |
| 85 | T/G | IKZF2 | loss | ELK4_1 | 0.001 | 0.003 | 0.050 |
| 86 | T/G | TFAP2A | loss | ETS_1 | 0.002 | 0.004 | 0.249 |
| 87 | T/G | AHR | loss | ETS1_3 | 0.002 | 0.005 | 0.300 |
| 88 | T/G | EGR1 | loss | ZNF143_2 | 0.002 | 0.036 | 0.520 |
| 89 | T/G | STAT1 | loss | STAT6_2 | 0.002 | 0.009 | 0.531 |
| 90 | T/G | FEV | loss | FEV_2 | 0.002 | 0.001 | 0.079 |
| 91 | T/G | PAX5 | loss | MA0028.1 | 0.002 | 0.005 | 0.178 |
| 92 | T/G | BARHL2 | loss | NFATC2_1 | 0.003 | 0.015 | 0.434 |
| 93 | T/G | AR | loss | ELF5_3 | 0.003 | 0.002 | 0.071 |
| 94 | T/G | NFE2L2 | loss | GABP_1 | 0.003 | 0.029 | 0.278 |
| 95 | T/G | TCF12 | loss | ITGB2_1 | 0.003 | 0.004 | 0.071 |
| 96 | T/G | E2F1 | loss | SPDEF_1 | 0.003 | 0.006 | 0.126 |
| 97 | T/G | KLF13 | loss | STAT_disc3 | 0.004 | 0.024 | 0.339 |
| 98 | T/G | SCRT1 | loss | IRF_1 | 0.004 | 0.012 | 0.302 |
| 99 | T/G | LBX2 | loss | NFAT_2 | 0.004 | 0.007 | 0.267 |
| 100 | T/G | SMAD | loss | MA0136.1 | 0.004 | 0.010 | 0.264 |
| 101 | T/G | IKZF1 | loss | FOXJ3_5 | 0.004 | 0.021 | 0.581 |
| 102 | T/G | IRF5 | loss | ELF5_1 | 0.004 | 0.007 | 0.329 |
| 103 | T/G | E2F1 | loss | RXRA_disc4 | 0.004 | 0.035 | 0.431 |
| 104 | T/G | RARA | loss | STAT_1 | 0.004 | 0.021 | 0.227 |
| 105 | T/G | PAX5 | loss | SRY_1 | 0.004 | 0.006 | 0.811 |
| 106 | T/G | ELK1 | loss | MA0473.1 | 0.004 | 0.003 | 0.064 |
| 107 | T/G | SIX5 | loss | ETS1_1 | 0 | 0.0000004 | 0.061 |
| 108 | T/G | REL | loss | ELF5_2 | 0.00001 | 0.00004 | 0.782 |
| 109 | T/G | PAX2 | loss | MA0081.1 | 0.0001 | 0.003 | 0.114 |
| 110 | T/G | STAT1 | loss | EHF_1 | 0.0002 | 0.001 | 0.099 |
| 111 | T/G | MEF2 | loss | STAT5A_2 | 0.0002 | 0.001 | 0.132 |
| 112 | T/G | NFATC1 | loss | ELF4_1 | 0.0002 | 0.004 | 0.906 |
| 113 | T/G | TFAP2C | loss | ELF3_1 | 0.0003 | 0.001 | 0.055 |
| 114 | T/G | ZBTB7A | loss | SPI1_1 | 0.0003 | 0.005 | 0.106 |
| 115 | T/G | MYB | loss | ELK1_2 | 0.0004 | 0.002 | 0.180 |
| 116 | T/G | E2F1 | loss | SPIB_1 | 0.0004 | 0.002 | 0.756 |
| 117 | T/G | RELA | loss | ETS_2 | 0.0004 | 0.002 | 0.591 |
| 118 | T/G | NFATC2 | loss | MA0080.3 | 0.001 | 0.017 | 0.399 |
| 119 | T/G | NR3C1 | loss | MA0062.2 | 0.001 | 0.002 | 0.089 |
| 120 | T/G | ETV3 | loss | MYC_disc6 | 0.001 | 0.012 | 0.157 |
| 121 | T/G | SPIC | loss | ETV4_2 | 0.001 | 0.001 | 0.313 |
| 122 | T/G | IRF1 | loss | ELK1_3 | 0.001 | 0.004 | 0.934 |
| 123 | T/G | MYC | loss | ETV6_2 | 0.001 | 0.005 | 0.989 |
| 124 | T/G | E2F1 | loss | ETS1_4 | 0.001 | 0.001 | 0.147 |
| 125 | T/G | FEV | loss | ETS1_2 | 0.001 | 0.004 | 0.118 |
| 126 | T/G | HNF4A | loss | SPDEF_5 | 0.001 | 0.002 | 0.694 |
| 127 | T/G | IRX3 | loss | ELK4_1 | 0.001 | 0.003 | 0.050 |
| 128 | T/G | AP1 | loss | ETS_1 | 0.002 | 0.004 | 0.249 |
| 129 | T/G | HESX1 | loss | ETS1_3 | 0.002 | 0.005 | 0.300 |
| 130 | T/G | EHF | loss | ZNF143_2 | 0.002 | 0.036 | 0.520 |
| 131 | T/G | RARB | loss | STAT6_2 | 0.002 | 0.009 | 0.531 |
| 132 | T/G | Arnt::Ahr | loss | FEV_2 | 0.002 | 0.001 | 0.079 |
|  | G^8^ | coronary vasculature morphogenesis (GO:0060977; FDR=**0.02**);  vasculogenesis (GO:0001570; FDR=**0.007**) | | | | | |
|  | T^9^ | negative regulation of vascular associated smooth muscle cell differentiation  (GO:1905064; FDR=**0.033**);  positive regulation of leukocyte adhesion to vascular endothelial cell (GO:1904996; FDR=**0.0097**);  positive regulation of interleukin-12 production (GO:0032735; FDR=**0.038**);  cellular response to hydrogen peroxide (GO:0070301; FDR=**0.008**);  regulation of transcription from RNA polymerase II promoter in response to hypoxia (GO:0061418; FDR=**0.047**);  apoptotic process (GO:0006915; FDR=**0.02**);  positive regulation of apoptotic process (GO:0043065; FDR=**0.007**) | | | | | |
| 1 – reference (Ref) / alternative (SNP) allele;  2 – TF - transcription factor;  3 – binding of TF to the reference (LOSS) / alternative (GAIN) allele;  4 – binding sites with high affinity for TF;  5 – p value statistically confirming the potential gain or loss of function of the genomic region with SNP in terms of transcription factor binding;  6 – p-value for assessing the binding of TF to the Ref allele;  7 – p-value for assessing the binding of TF to the SNP allele;  8 – biological processes pathogenetically significant for IS, in which TFs that bind to the alternative allele are jointly involved (data from the Gene Ontology resource; http://geneontology.org/);  9 – biological processes pathogenetically significant for AI, in which TFs that bind to the reference allele are jointly involved (data from the Gene Ontology resource; http://geneontology.org/) | | | | | | | |

Supplementary Table 8: Analysis of the effect of rs4644832 *SERF2* on the binding of DNA to transcription factors.

| № | Ref/SNP allele^1^ | TF^2^ | GAIN  /LOSS^3^ | Motif^4^ | P-Value SNP impact^5^ | P-Value Ref^6^ | P-Value SNP^7^ |
| --- | --- | --- | --- | --- | --- | --- | --- |
| 1 | G/A | RHOXF1 | gain | RHOXF1_7 | 0.005 | 0.579 | 0.038 |
| 2 | G/A | TATA | gain | TATA_disc4 | 0.006 | 0.092 | 0.008 |
| 3 | G/A | MZF1 | gain | MZF1_2 | 0.006 | 0.750 | 0.035 |
| 4 | G/A | FOXC1 | gain | FOXC1_2 | 0.007 | 0.388 | 0.013 |
| 5 | G/A | RREB1 | gain | RREB1_1 | 0.014 | 0.114 | 0.028 |
| 6 | G/A | HDAC2 | gain | HDAC2_disc3 | 0.016 | 0.317 | 0.039 |
| 7 | G/A | RFX1 | gain | RFX1_2 | 0.017 | 0.257 | 0.024 |
| 8 | G/A | PAX9 | gain | PAX9_1 | 0.019 | 0.279 | 0.024 |
| 9 | G/A | SETDB1 | gain | SETDB1_disc2 | 0.021 | 0.388 | 0.030 |
| 10 | G/A | SPZ1 | gain | SPZ1_1 | 0.023 | 0.191 | 0.040 |
| 11 | G/A | FOXC1 | gain | MA0032.1 | 0.024 | 0.386 | 0.023 |
| 12 | G/A | EWSR1::FLI1 | gain | EWSR1::FLI1_1 | 0.027 | 0.177 | 0.049 |
| 13 | G/A | ZNF784 | gain | ZNF784_1 | 0.031 | 0.067 | 0.009 |
| 14 | G/A | NR3C1 | loss | NR3C1_disc6 | 0 | 0 | 0.169 |
| 15 | G/A | YY1 | loss | YY1_disc4 | 0 | 0 | 0.196 |
| 16 | G/A | YY1 | loss | YY1_disc5 | 0 | 0 | 0.294 |
| 17 | G/A | ESRRA | loss | ESRRA_disc4 | 0.000004 | 0 | 0.113 |
| 18 | G/A | REST | loss | REST_disc8 | 0.0001 | 0.00004 | 0.095 |
| 19 | G/A | RUNX | loss | RUNX_1 | 0.0002 | 0.002 | 0.111 |
| 20 | G/A | RUNX1 | loss | MA0002.2 | 0.0002 | 0.002 | 0.287 |
| 21 | G/A | RUNX1 | loss | RUNX1_9 | 0.0003 | 0.001 | 0.320 |
| 22 | G/A | RUNX2 | loss | RUNX2_5 | 0.002 | 0.010 | 0.238 |
| 23 | G/A | BCL | loss | BCL_disc9 | 0.002 | 0.002 | 0.056 |
| 24 | G/A | SP2 | loss | SP2_disc1 | 0.003 | 0.020 | 0.333 |
| 25 | G/A | ZBED1 | loss | ZBED1_1 | 0.004 | 0.025 | 0.731 |
| 26 | G/A | REST | loss | REST_disc4 | 0.004 | 0.023 | 0.372 |
| 27 | G/A | REST | loss | REST_disc9 | 0.005 | 0.004 | 0.090 |
| 28 | G/A | TFAP2A | loss | TFAP2A_4 | 0.005 | 0.034 | 0.457 |
| 29 | G/A | TFAP2A | loss | TFAP2A_1 | 0.005 | 0.034 | 0.457 |
| 30 | G/A | CTCF | loss | CTCF_disc4 | 0.006 | 0.021 | 0.477 |
| 31 | G/A | GLI2 | loss | GLI2_2 | 0.006 | 0.007 | 0.115 |
| 32 | G/A | RUNX1 | loss | RUNX1_7 | 0.007 | 0.009 | 0.151 |
| 33 | G/A | RUNX | loss | RUNX_2 | 0.007 | 0.040 | 0.511 |
| 34 | G/A | PLAG1 | loss | PLAG1_1 | 0.009 | 0.034 | 0.226 |
| 35 | G/A | BCL | loss | BCL_disc10 | 0.009 | 0.047 | 0.398 |
| 36 | G/A | NFE2 | loss | NFE2_disc4 | 0.010 | 0.028 | 0.224 |
| 37 | G/A | TBX2 | loss | TBX2_1 | 0.011 | 0.006 | 0.051 |
| 38 | G/A | HIF1A::ARNT | loss | HIF1A::ARNT_1 | 0.012 | 0.032 | 0.554 |
| 39 | G/A | E2F4 | loss | E2F4_3 | 0.015 | 0.039 | 0.207 |
| 40 | G/A | RUNX2 | loss | RUNX2_3 | 0.023 | 0.002 | 0.059 |
| 41 | G/A | TBX5 | loss | TBX5_5 | 0.026 | 0.036 | 0.185 |
| 42 | G/A | POU2F2 | loss | POU2F2_disc2 | 0.027 | 0.016 | 0.078 |
| 43 | G/A | BCL | loss | BCL_disc3 | 0.028 | 0.033 | 0.286 |
| 44 | G/A | SIN3A | loss | SIN3A_disc7 | 0.029 | 0.015 | 0.080 |
| 45 | G/A | ESR1 | loss | ESR1_3 | 0.031 | 0.012 | 0.054 |
| 46 | G/A | RUNX3 | loss | RUNX3_2 | 0.034 | 0.010 | 0.070 |
| 47 | G/A | TBX4 | loss | TBX4_2 | 0.035 | 0.029 | 0.137 |
| 48 | G/A | ZIC1 | loss | ZIC1_1 | 0.035 | 0.030 | 0.213 |
| 49 | G/A | ELF1 | loss | ELF1_disc3 | 0.037 | 0.048 | 0.182 |
| 50 | G/A | TATA | loss | TATA_disc10 | 0.041 | 0.039 | 0.240 |
| 51 | G/A | ZSCAN16 | loss | NR3C1_disc6 | 0 | 0 | 0.169 |
| 52 | G/A | SMAD | loss | YY1_disc4 | 0 | 0 | 0.196 |
| 53 | G/A | RREB1 | loss | YY1_disc5 | 0 | 0 | 0.294 |
| A^8^ | | - | | | | | |
| G^9^ | | regulation of smoothened signaling pathway (GO:0008589; FDR=**0.02**) | | | | | |
| 1 – reference (Ref) / alternative (SNP) allele;  2 – TF - transcription factor;  3 – binding of TF to the reference (LOSS) / alternative (GAIN) allele;  4 – binding sites with high affinity for TF;  5 – p value statistically confirming the potential gain or loss of function of the genomic region with SNP in terms of transcription factor binding;  6 – p-value for assessing the binding of TF to the Ref allele;  7 – p-value for assessing the binding of TF to the SNP allele;  8 – biological processes pathogenetically significant for IS, in which TFs that bind to the alternative allele are jointly involved (data from the Gene Ontology resource; http://geneontology.org/);  9 – biological processes pathogenetically significant for AI, in which TFs that bind to the reference allele are jointly involved (data from the Gene Ontology resource; http://geneontology.org/) | | | | | | | |

Supplementary Table 9: Analysis of the effect of rs2901077 *C19orf53* on the binding of DNA to transcription factors.

| № | Ref/SNP allele^1^ | TF^2^ | GAIN  /LOSS^3^ | Motif^4^ | P-Value SNP impact^5^ | P-Value Ref^6^ | P-Value SNP^7^ |
| --- | --- | --- | --- | --- | --- | --- | --- |
| 1 | C/T | ZBTB14 | gain | ZBTB14_1 | 0 | 0.092 | 0 |
| 2 | C/T | TATA | gain | TATA_disc1 | 0.002 | 0.275 | 0.014 |
| 3 | C/T | MYCN | gain | MYCN_2 | 0.002 | 0.749 | 0.011 |
| 4 | C/T | ZNF354C | gain | ZNF354C_1 | 0.003 | 0.365 | 0.018 |
| 5 | C/T | ARNT | gain | ARNT_1 | 0.003 | 0.074 | 0.002 |
| 6 | C/T | ZBTB3 | gain | ZBTB3_1 | 0.004 | 0.465 | 0.034 |
| 7 | C/T | MYC | gain | MYC_disc2 | 0.005 | 0.281 | 0.011 |
| 8 | C/T | RAD21 | gain | RAD21_disc5 | 0.015 | 0.358 | 0.041 |
| 9 | C/T | ZNF354C | gain | MA0130.1 | 0.018 | 0.374 | 0.029 |
| 10 | C/T | REST | gain | REST_disc7 | 0.018 | 0.193 | 0.017 |
| 11 | C/T | SIX5 | gain | ZBTB14_1 | 0 | 0.092 | 0 |
| 12 | C/T | ZNF8 | gain | TATA_disc1 | 0.002 | 0.275 | 0.014 |
| 13 | C/T | ELK1 | gain | MYCN_2 | 0.002 | 0.749 | 0.011 |
| 14 | C/T | NFIB | gain | ZNF354C_1 | 0.003 | 0.365 | 0.018 |
| 15 | C/T | ELF1 | gain | ARNT_1 | 0.003 | 0.074 | 0.002 |
| 16 | C/T | NFIA | gain | ZBTB3_1 | 0.004 | 0.465 | 0.034 |
| 17 | C/T | SRF | loss | SRF_disc2 | 0 | 0 | 0.126 |
| 18 | C/T | ETS | loss | ETS_disc7 | 0 | 0.0000002 | 0.147 |
| 19 | C/T | NFE2 | loss | NFE2_disc3 | 0 | 0.0000002 | 0.197 |
| 20 | C/T | YY1 | loss | YY1_disc4 | 0.000002 | 0.001 | 0.213 |
| 21 | C/T | CHD2 | loss | CHD2_disc3 | 0.00002 | 0.00003 | 0.086 |
| 22 | C/T | ELK1 | loss | ELK1_2 | 0.00004 | 0.0003 | 0.104 |
| 23 | C/T | ELF1 | loss | ELF1_disc3 | 0.001 | 0.002 | 0.168 |
| 24 | C/T | SPDEF | loss | SPDEF_6 | 0.001 | 0.006 | 0.243 |
| 25 | C/T | ZBTB33 | loss | ZBTB33_disc2 | 0.003 | 0.007 | 0.209 |
| 26 | C/T | NR2C2 | loss | NR2C2_disc3 | 0.003 | 0.008 | 0.108 |
| 27 | C/T | FLI1 | loss | FLI1_4 | 0.004 | 0.025 | 0.461 |
| 28 | C/T | ETV5 | loss | ETV5_1 | 0.004 | 0.004 | 0.135 |
| 29 | C/T | HDAC2 | loss | HDAC2_disc4 | 0.004 | 0.018 | 0.782 |
| 30 | C/T | MYCN | loss | MYCN_1 | 0.004 | 0.008 | 0.106 |
| 31 | C/T | BHLHE40 | loss | BHLHE40_disc2 | 0.005 | 0.021 | 0.253 |
| 32 | C/T | SPDEF | loss | SPDEF_2 | 0.005 | 0.016 | 0.684 |
| 33 | C/T | ELK4 | loss | ELK4_2 | 0.007 | 0.008 | 0.217 |
| 34 | C/T | ERG | loss | ERG_3 | 0.008 | 0.016 | 0.704 |
| 35 | C/T | CTCF | loss | CTCF_disc9 | 0.008 | 0.036 | 0.304 |
| 36 | C/T | YY1 | loss | YY1_disc3 | 0.008 | 0.010 | 0.133 |
| 37 | C/T | ELK1 | loss | ELK1_5 | 0.009 | 0.017 | 0.304 |
| 38 | C/T | FLI1 | loss | FLI1_1 | 0.010 | 0.008 | 0.209 |
| 39 | C/T | EP300 | loss | EP300_disc8 | 0.010 | 0.005 | 0.064 |
| 40 | C/T | SPDEF | loss | SPDEF_5 | 0.010 | 0.006 | 0.157 |
| 41 | C/T | REST | loss | REST_disc8 | 0.011 | 0.024 | 0.170 |
| 42 | C/T | ETS1 | loss | ETS1_4 | 0.011 | 0.016 | 0.253 |
| 43 | C/T | RAD21 | loss | RAD21_disc8 | 0.012 | 0.040 | 0.271 |
| 44 | C/T | BCL | loss | BCL_disc1 | 0.012 | 0.012 | 0.264 |
| 45 | C/T | ETS1 | loss | ETS1_6 | 0.012 | 0.008 | 0.179 |
| 46 | C/T | FLI1 | loss | FLI1_3 | 0.012 | 0.015 | 0.264 |
| 47 | C/T | ETV1 | loss | ETV1_1 | 0.013 | 0.008 | 0.131 |
| 48 | C/T | E2F1 | loss | E2F1_11 | 0.013 | 0.017 | 0.117 |
| 49 | C/T | ELK1 | loss | ELK1_4 | 0.013 | 0.013 | 0.194 |
| 50 | C/T | ERG | loss | ERG_1 | 0.013 | 0.014 | 0.224 |
| 51 | C/T | ZBTB7A | loss | ZBTB7A_1 | 0.014 | 0.031 | 0.251 |
| 52 | C/T | SPDEF | loss | SPDEF_1 | 0.015 | 0.036 | 0.425 |
| 53 | C/T | E2F1 | loss | E2F1_15 | 0.015 | 0.038 | 0.417 |
| 54 | C/T | ELK3 | loss | ELK3_2 | 0.016 | 0.016 | 0.234 |
| 55 | C/T | ELK3 | loss | ELK3_1 | 0.017 | 0.011 | 0.202 |
| 56 | C/T | FEV | loss | FEV_2 | 0.018 | 0.017 | 0.238 |
| 57 | C/T | GABPA | loss | SRF_disc2 | 0 | 0 | 0.126 |
| 58 | C/T | MTF1 | loss | ETS_disc7 | 0 | 0.0000002 | 0.147 |
| 59 | C/T | MLX | loss | NFE2_disc3 | 0 | 0.0000003 | 0.197 |
| 60 | C/T | TATA | loss | YY1_disc4 | 0.000002 | 0.001 | 0.213 |
| 61 | C/T | ETV2 | loss | CHD2_disc3 | 0.00002 | 0.00003 | 0.086 |
| 62 | C/T | E2F1 | loss | ELK1_2 | 0.00004 | 0.0003 | 0.104 |
| 63 | C/T | ETV4 | loss | ELF1_disc3 | 0.001 | 0.002 | 0.168 |
| 64 | C/T | HEY2 | loss | SPDEF_6 | 0.001 | 0.006 | 0.243 |
| 65 | C/T | E2F1 | loss | ZBTB33_disc2 | 0.003 | 0.007 | 0.209 |
| 66 | C/T | KLF12 | loss | NR2C2_disc3 | 0.003 | 0.008 | 0.108 |
| 67 | C/T | GABPA | loss | FLI1_4 | 0.004 | 0.025 | 0.461 |
| 68 | C/T | E2F1 | loss | ETV5_1 | 0.004 | 0.004 | 0.135 |
| 69 | C/T | MYC::MAX | loss | HDAC2_disc4 | 0.004 | 0.018 | 0.782 |
|  | T^8^ | - | | | | | |
|  | C^9^ | cardiac vascular smooth muscle cell differentiation (GO:0060947; FDR=**0.019**);  dorsal aorta morphogenesis (GO:0035912; FDR=**0.019**);  response to oxygen levels (GO:0070482; FDR=**0.035**) | | | | | |
| 1 – reference (Ref) / alternative (SNP) allele;  2 – TF - transcription factor;  3 – binding of TF to the reference (LOSS) / alternative (GAIN) allele;  4 – binding sites with high affinity for TF;  5 – p value statistically confirming the potential gain or loss of function of the genomic region with SNP in terms of transcription factor binding;  6 – p-value for assessing the binding of TF to the Ref allele;  7 – p-value for assessing the binding of TF to the SNP allele;  8 – biological processes pathogenetically significant for IS, in which TFs that bind to the alternative allele are jointly involved (data from the Gene Ontology resource; http://geneontology.org/);  9 – biological processes pathogenetically significant for AI, in which TFs that bind to the reference allele are jointly involved (data from the Gene Ontology resource; http://geneontology.org/) | | | | | | | |

Supplementary Table 10: Analysis of the effect of rs346157 *C19orf53* on the binding of DNA to transcription factors.

| № | Ref/SNP allele^1^ | TF^2^ | GAIN  /LOSS^3^ | Motif^4^ | P-Value SNP impact^5^ | P-Value Ref^6^ | P-Value SNP^7^ |
| --- | --- | --- | --- | --- | --- | --- | --- |
| 1 | A/G | SRF | gain | SRF_disc2 | 0 | 0.055 | <0.001 |
| 2 | A/G | ZNF143 | gain | ZNF143_disc4 | 0 | 0.142 | <0.001 |
| 3 | A/G | HDAC2 | gain | HDAC2_disc4 | 0.0000002 | 0.526 | <0.001 |
| 4 | A/G | YY1 | gain | YY1_disc5 | 0.000001 | 0.243 | <0.001 |
| 5 | A/G | MAZ | gain | MAZ_1 | 0.001 | 0.125 | 0.008 |
| 6 | A/G | ELF1 | gain | ELF1_disc2 | 0.003 | 0.172 | 0.010 |
| 7 | A/G | E2F | gain | E2F_disc8 | 0.003 | 0.133 | 0.005 |
| 8 | A/G | RARG | gain | RARG_9 | 0.004 | 0.568 | 0.035 |
| 9 | A/G | CREB3L2 | gain | CREB3L2_2 | 0.007 | 0.412 | 0.018 |
| 10 | A/G | RAD21 | gain | RAD21_disc8 | 0.011 | 0.214 | 0.030 |
| 11 | A/G | PLAG1 | gain | MA0163.1 | 0.012 | 0.107 | 0.019 |
| 12 | A/G | REST | gain | REST_disc5 | 0.014 | 0.281 | 0.041 |
| 13 | A/G | REST | gain | SRF_disc2 | 0 | 0.055 | 0 |
| 14 | A/G | FOS | gain | ZNF143_disc4 | 0 | 0.142 | 0.0000001 |
| 15 | A/G | BDP1 | gain | HDAC2_disc4 | 0.0000002 | 0.526 | 0.00001 |
| 16 | A/G | RAD21 | gain | YY1_disc5 | 0.000001 | 0.243 | 0.0000005 |
| 17 | A/G | GATA | gain | MAZ_1 | 0.001 | 0.125 | 0.0080 |
| 18 | A/G | TFCP2 | gain | ELF1_disc2 | 0.003 | 0.172 | 0.014 |
| 19 | A/G | JUN (var.2) | gain | E2F_disc8 | 0.003 | 0.133 | 0.005 |
| 20 | A/G | AP1 | gain | RARG_9 | 0.004 | 0.568 | 0.035 |
| 21 | A/G | STAT | gain | CREB3L2_2 | 0.007 | 0.412 | 0.018 |
| 22 | A/G | E2F1 | gain | RAD21_disc8 | 0.011 | 0.214 | 0.030 |
| 23 | A/G | MAF | loss | MAF_2 | 0.0001 | 0.001 | 0.893 |
| 24 | A/G | NFE2L2 | loss | NFE2L2_2 | 0.001 | 0.019 | 0.307 |
| 25 | A/G | WT1 | loss | WT1_1 | 0.002 | 0.01 | 0.408 |
| 26 | A/G | ATF4 | loss | ATF4_2 | 0.002 | 0.023 | 0.851 |
| 27 | A/G | AP1 | loss | AP1_7 | 0.003 | 0.022 | 0.703 |
| 28 | A/G | CREB1 | loss | CREB1_2 | 0.003 | 0.012 | 0.342 |
| 29 | A/G | REST | loss | REST_3 | 0.004 | 0.023 | 0.115 |
| 30 | A/G | SP1 | loss | SP1_disc3 | 0.004 | 0.02 | 0.443 |
| 31 | A/G | EP300 | loss | EP300_disc2 | 0.005 | 0.033 | 0.823 |
| 32 | A/G | AP1 | loss | AP1_10 | 0.006 | 0.031 | 1 |
| 33 | A/G | NHLH1 | loss | NHLH1_3 | 0.006 | 0.015 | 0.418 |
| 34 | A/G | BACH2 | loss | BACH2_1 | 0.006 | 0.011 | 0.264 |
| 35 | A/G | JDP2 | loss | JDP2_2 | 0.006 | 0.042 | 1 |
| 36 | A/G | ATF2 | loss | ATF2_2 | 0.006 | 0.014 | 0.236 |
| 37 | A/G | HMGN3 | loss | HMGN3_disc1 | 0.007 | 0.01 | 0.131 |
| 38 | A/G | CREB1 | loss | CREB1_9 | 0.007 | 0.02 | 0.392 |
| 39 | A/G | PAX4 | loss | PAX4_3 | 0.007 | 0.038 | 0.98 |
| 40 | A/G | TFAP2 | loss | TFAP2_disc1 | 0.008 | 0.012 | 0.146 |
| 41 | A/G | CACBP | loss | CACBP_1 | 0.008 | 0.010 | 0.132 |
| 42 | A/G | RHOXF1 | loss | RHOXF1_3 | 0.008 | 0.028 | 0.531 |
| 43 | A/G | RHOXF1 | loss | RHOXF1_5 | 0.008 | 0.017 | 0.286 |
| 44 | A/G | BCL | loss | BCL_disc2 | 0.010 | 0.028 | 0.242 |
| 45 | A/G | AP1 | loss | AP1_9 | 0.010 | 0.049 | 1 |
| 46 | A/G | EP300 | loss | EP300_disc1 | 0.010 | 0.006 | 0.096 |
| 47 | A/G | ESRRA | loss | ESRRA_6 | 0.011 | 0.008 | 0.120 |
| 48 | A/G | RXRA | loss | RXRA_disc4 | 0.011 | 0.023 | 0.188 |
| 49 | A/G | BACH1 | loss | BACH1_1 | 0.011 | 0.024 | 0.208 |
| 50 | A/G | JUNB | loss | MA0490.1 | 0.012 | 0.024 | 0.208 |
| 51 | A/G | PRDM1 | loss | PRDM1_disc2 | 0.013 | 0.031 | 0.227 |
| 52 | A/G | FOSL2 | loss | MA0478.1 | 0.013 | 0.021 | 0.217 |
| 53 | A/G | TAL1 | loss | TAL1_disc1 | 0.014 | 0.018 | 0.188 |
| 54 | A/G | BATF | loss | BATF_disc1 | 0.015 | 0.026 | 0.197 |
| 55 | A/G | NHLH1 | loss | MA0048.1 | 0.015 | 0.022 | 0.184 |
| 56 | A/G | CACD | loss | CACD_1 | 0.015 | 0.028 | 0.202 |
| 57 | A/G | HSF1 | loss | HSF1_2 | 0.016 | 0.045 | 0.255 |
| 58 | A/G | Bach1::Mafk | loss | MA0591.1 | 0.016 | 0.010 | 0.068 |
| 59 | A/G | CREB1 | loss | CREB1_6 | 0.018 | 0.030 | 0.386 |
| 60 | A/G | FOSL1 | loss | MA0477.1 | 0.019 | 0.019 | 0.172 |
| 61 | A/G | EGR4 | loss | MAF_2 | 0.000 | 0.001 | 0.893 |
| 62 | A/G | E2F7 | loss | NFE2L2_2 | 0.001 | 0.019 | 0.307 |
| 63 | A/G | NR3C1 | loss | WT1_1 | 0.002 | 0.010 | 0.408 |
| 64 | A/G | PAX5 | loss | ATF4_2 | 0.002 | 0.023 | 0.851 |
| 65 | A/G | JUN::FOS | loss | AP1_7 | 0.003 | 0.022 | 0.703 |
| 66 | A/G | MEF2 | loss | CREB1_2 | 0.003 | 0.012 | 0.342 |
| 67 | A/G | JUND | loss | REST_3 | 0.004 | 0.023 | 0.115 |
| 68 | A/G | E2F1 | loss | SP1_disc3 | 0.004 | 0.020 | 0.443 |
| 69 | A/G | ZNF143 | loss | EP300_disc2 | 0.005 | 0.033 | 0.823 |
| 70 | A/G | TCF7L2 | loss | AP1_10 | 0.006 | 0.031 | 1.000 |
| 71 | A/G | E2F6 | loss | NHLH1_3 | 0.006 | 0.015 | 0.418 |
| 72 | A/G | GLI2 | loss | BACH2_1 | 0.006 | 0.011 | 0.264 |
| 73 | A/G | JDP2 | loss | JDP2_2 | 0.006 | 0.042 | 1.000 |
| 74 | A/G | CREB1 | loss | ATF2_2 | 0.006 | 0.014 | 0.236 |
| 75 | A/G | PAX5 | loss | HMGN3_disc1 | 0.007 | 0.010 | 0.131 |
| 76 | A/G | ELK1 | loss | CREB1_9 | 0.007 | 0.020 | 0.392 |
| 77 | A/G | TRIM28 | loss | PAX4_3 | 0.007 | 0.038 | 0.980 |
| 78 | A/G | IRF | loss | TFAP2_disc1 | 0.008 | 0.012 | 0.146 |
| 79 | A/G | BHLHE40 | loss | CACBP_1 | 0.008 | 0.010 | 0.132 |
| 80 | A/G | EGR3 | loss | RHOXF1_3 | 0.008 | 0.028 | 0.531 |
| 81 | A/G | POU5F1 | loss | RHOXF1_5 | 0.008 | 0.017 | 0.286 |
| 82 | A/G | SPDEF | loss | BCL_disc2 | 0.010 | 0.028 | 0.242 |
| 83 | A/G | Pou5f1::Sox2 | loss | AP1_9 | 0.010 | 0.049 | 1.000 |
| 84 | A/G | MAFB | loss | EP300_disc1 | 0.010 | 0.006 | 0.096 |
| 85 | A/G | MAFK | loss | ESRRA_6 | 0.011 | 0.008 | 0.120 |
| G^8^ | | cellular response to reactive oxygen species (GO:0034614; FDR=**0.035**);  response to hypoxia (GO:0001666; FDR=**0.02**) | | | | | |
| A^9^ | | - | | | | | |
| 1 – reference (Ref) / alternative (SNP) allele;  2 – TF - transcription factor;  3 – binding of TF to the reference (LOSS) / alternative (GAIN) allele;  4 – binding sites with high affinity for TF;  5 – p value statistically confirming the potential gain or loss of function of the genomic region with SNP in terms of transcription factor binding;  6 – p-value for assessing the binding of TF to the Ref allele;  7 – p-value for assessing the binding of TF to the SNP allele;  8 – biological processes pathogenetically significant for IS, in which TFs that bind to the alternative allele are jointly involved (data from the Gene Ontology resource; [http://geneontology.org/](http://geneontology,org/));  9 – biological processes pathogenetically significant for AI, in which TFs that bind to the reference allele are jointly involved (data from the Gene Ontology resource; [http://geneontology.org/](http://geneontology,org/)) | | | | | | | |

Supplementary Table 11: Analysis of the effect of rs11666524 *C19orf53* on the binding of DNA to transcription factors.

| № | Ref/SNP allele ^1^ | ТФ^2^ | GAIN  /LOSS^3^ | Motif^4^ | P-Value SNP impact^5^ | P-Value Ref^6^ | P-Value SNP^7^ |
| --- | --- | --- | --- | --- | --- | --- | --- |
| 1 | G/A | REST | gain | REST_disc1 | 0.001 | 0.461 | 0.011 |
| 2 | G/A | SMC3 | gain | SMC3_disc2 | 0.002 | 0.059 | 0.005 |
| 3 | G/A | MAF | gain | MAF_1 | 0.003 | 0.099 | 0.011 |
| 4 | G/A | SMC3 | gain | SMC3_disc4 | 0.004 | 0.056 | 0.004 |
| 5 | G/A | TCF4 | gain | TCF4_1 | 0.005 | 0.507 | 0.022 |
| 6 | G/A | RXRA | gain | RXRA_disc1 | 0.006 | 0.348 | 0.025 |
| 7 | G/A | TCF12 | gain | TCF12_disc1 | 0.006 | 0.455 | 0.011 |
| 8 | G/A | RAD21 | gain | RAD21_disc10 | 0.009 | 0.125 | 0.010 |
| 9 | G/A | Stat4 | gain | MA0518.1 | 0.011 | 0.144 | 0.011 |
| 10 | G/A | E2F7 | gain | E2F7_1 | 0.011 | 0.123 | 0.007 |
| 11 | G/A | SREBF1 | gain | SREBF1_3 | 0.013 | 0.383 | 0.043 |
| 12 | G/A | TAL1 | gain | TAL1_disc1 | 0.014 | 0.295 | 0.027 |
| 13 | G/A | ID4 | gain | ID4_1 | 0.020 | 0.307 | 0.026 |
| 14 | G/A | GCM1 | gain | GCM1_2 | 0.025 | 0.272 | 0.027 |
| 15 | G/A | GCM1 | gain | GCM1_1 | 0.026 | 0.343 | 0.045 |
| 16 | G/A | MYF | gain | MYF_1 | 0.030 | 0.162 | 0.025 |
| 17 | G/A | CTCF | gain | CTCF_disc6 | 0.031 | 0.309 | 0.041 |
| 18 | G/A | CHD2 | gain | CHD2_disc1 | 0.046 | 0.239 | 0.038 |
| 19 | G/A | HNF4 | gain | HNF4_disc2 | 0.046 | 0.264 | 0.041 |
| 20 | G/A | ELF1 | loss | ELF1_disc3 | 0.0002 | 0.001 | 0.251 |
| 21 | G/A | NFATC1 | loss | NFATC1_3 | 0.001 | 0.007 | 0.235 |
| 22 | G/A | E2F1 | loss | E2F1_13 | 0.001 | 0.013 | 0.324 |
| 23 | G/A | ERF | loss | ERF_1 | 0.002 | 0.010 | 0.649 |
| 24 | G/A | NR3C1 | loss | NR3C1_6 | 0.003 | 0.014 | 0.205 |
| 25 | G/A | ZNF784 | loss | ZNF784_1 | 0.003 | 0.011 | 0.542 |
| 26 | G/A | ZBTB33 | loss | ZBTB33_disc2 | 0.003 | 0.009 | 0.258 |
| 27 | G/A | E2F1 | loss | E2F1_6 | 0.005 | 0.007 | 0.270 |
| 28 | G/A | E2F1 | loss | E2F1_18 | 0.005 | 0.019 | 0.359 |
| 29 | G/A | E2F1 | loss | E2F1_8 | 0.006 | 0.010 | 0.315 |
| 30 | G/A | ETS | loss | ETS_disc4 | 0.007 | 0.027 | 0.345 |
| 31 | G/A | ELK1 | loss | ELK1_6 | 0.008 | 0.011 | 0.107 |
| 32 | G/A | SRF | loss | SRF_disc2 | 0.009 | 0.043 | 0.300 |
| 33 | G/A | AP1 | loss | AP1_disc7 | 0.010 | 0.033 | 0.260 |
| 34 | G/A | ELK4 | loss | ELK4_1 | 0.011 | 0.012 | 0.165 |
| 35 | G/A | PTF1A | loss | PTF1A_1 | 0.012 | 0.017 | 0.160 |
| 36 | G/A | ATF3 | loss | ATF3_disc3 | 0.012 | 0.005 | 0.076 |
| 37 | G/A | SPDEF | loss | SPDEF_6 | 0.012 | 0.023 | 0.292 |
| 38 | G/A | AR | loss | AR_6 | 0.016 | 0.046 | 0.629 |
| 39 | G/A | TATA | loss | TATA_disc3 | 0.023 | 0.021 | 0.255 |
| 40 | G/A | NRF1 | loss | MA0506.1 | 0.031 | 0.021 | 0.200 |
| 41 | G/A | NR2C2 | loss | NR2C2_disc1 | 0.040 | 0.027 | 0.142 |
| 42 | G/A | CENPB | loss | CENPB_1 | 0.041 | 0.048 | 0.262 |
| 43 | G/A | FOXG1 | loss | FOXG1_4 | 0.047 | 0.027 | 0.151 |
| 44 | G/A | TCF3 | loss | TCF3_6 | 0.047 | 0.019 | 0.078 |
|  | A^8^ | - | | | | | |
|  | G^9^ | - | | | | | |
| 1 – reference (Ref) / alternative (SNP) allele;  2 – TF - transcription factor;  3 – binding of TF to the reference (LOSS) / alternative (GAIN) allele;  4 – binding sites with high affinity for TF;  5 – p value statistically confirming the potential gain or loss of function of the genomic region with SNP in terms of transcription factor binding;  6 – p-value for assessing the binding of TF to the Ref allele;  7 – p-value for assessing the binding of TF to the SNP allele;  8 – biological processes pathogenetically significant for IS, in which TFs that bind to the alternative allele are jointly involved (data from the Gene Ontology resource; http://geneontology.org/);  9 – biological processes pathogenetically significant for AI, in which TFs that bind to the reference allele are jointly involved (data from the Gene Ontology resource; http://geneontology.org/) | | | | | | | |

Supplementary Table 12: Analysis of the effect of rs7951676 *C11orf58* on the binding of DNA to transcription factors.

| № | Ref/SNP allele ^1^ | TF^2^ | GAIN  /LOSS^3^ | Motif^4^ | P-Value SNP impact^5^ | P-Value Ref^6^ | P-Value SNP^7^ |
| --- | --- | --- | --- | --- | --- | --- | --- |
| 1 | G/T | THAP1 | gain | THAP1_disc1 | 0.00006 | 0.169 | 0.003 |
| 2 | G/T | ZBTB6 | gain | ZBTB6_1 | 0.0004 | 0.074 | 0.001 |
| 3 | G/T | FOXB1 | gain | FOXB1_3 | 0.002 | 0.752 | 0.027 |
| 4 | G/T | YY1 | gain | YY1_disc1 | 0.003 | 0.253 | 0.010 |
| 5 | G/T | HDX | gain | HDX_1 | 0.004 | 0.511 | 0.030 |
| 6 | G/T | BCL | gain | BCL_disc3 | 0.006 | 0.055 | 0.002 |
| 7 | G/T | TBP | gain | MA0108.2 | 0.007 | 0.480 | 0.039 |
| 8 | G/T | TATA | gain | TATA_disc1 | 0.007 | 0.163 | 0.006 |
| 9 | G/T | HDAC2 | gain | HDAC2_disc2 | 0.010 | 0.513 | 0.040 |
| 10 | G/T | TBP | gain | TBP_5 | 0.010 | 0.482 | 0.041 |
| 11 | G/T | TBP | gain | TBP_2 | 0.010 | 0.482 | 0.041 |
| 12 | G/T | T | gain | T_3 | 0.011 | 0.375 | 0.041 |
| 13 | G/T | FOXC1 | gain | FOXC1_7 | 0.014 | 1.000 | 0.048 |
| 14 | G/T | TAL1::GATA1 | gain | MA0140.2 | 0.015 | 0.151 | 0.014 |
| 15 | G/T | FOXB1 | gain | FOXB1_4 | 0.029 | 0.337 | 0.037 |
| 16 | G/T | HLF | gain | HLF_4 | 0.029 | 0.400 | 0.046 |
| 17 | G/T | NR1H4 | gain | NR1H4_2 | 0.036 | 0.243 | 0.043 |
| 18 | G/T | NKX3-1 | gain | NKX3-1_2 | 0.041 | 0.509 | 0.049 |
| 19 | G/T | TAL1 | loss | TAL1_4 | 0 | 0.049 | 0.347 |
| 20 | G/T | POU2F1 | loss | POU2F1_6 | 0.001 | 0.002 | 0.083 |
| 21 | G/T | MYCN | loss | MYCN_1 | 0.002 | 0.009 | 0.212 |
| 22 | G/T | MYC::MAX | loss | MYC::MAX_3 | 0.002 | 0.007 | 0.146 |
| 23 | G/T | GLIS3 | loss | GLIS3_1 | 0.002 | 0.010 | 0.289 |
| 24 | G/T | ZBTB49 | loss | ZBTB49_1 | 0.003 | 0.039 | 0.372 |
| 25 | G/T | EBF1 | loss | EBF1_disc1 | 0.004 | 0.010 | 0.087 |
| 26 | G/T | AP1 | loss | AP1_disc9 | 0.006 | 0.010 | 0.136 |
| 27 | G/T | EBF1 | loss | MA0154.2 | 0.006 | 0.023 | 0.281 |
| 28 | G/T | RAD21 | loss | RAD21_disc2 | 0.007 | 0.026 | 0.265 |
| 29 | G/T | ATF3 | loss | ATF3_disc2 | 0.010 | 0.023 | 0.215 |
| 30 | G/T | CENPB | loss | CENPB_1 | 0.010 | 0.022 | 0.347 |
| 31 | G/T | MLXIPL | loss | MLXIPL_1 | 0.011 | 0.021 | 0.354 |
| 32 | G/T | MAX | loss | MA0058.2 | 0.013 | 0.019 | 0.179 |
| 33 | G/T | USF1 | loss | USF1_1 | 0.013 | 0.029 | 0.308 |
| 34 | G/T | USF2 | loss | MA0526.1 | 0.013 | 0.029 | 0.251 |
| 35 | G/T | USF2 | loss | USF2_1 | 0.018 | 0.020 | 0.238 |
| 36 | G/T | MAX | loss | MAX_2 | 0.019 | 0.026 | 0.286 |
| 37 | G/T | TFEB | loss | TFEB_1 | 0.019 | 0.024 | 0.243 |
| 38 | G/T | USF1 | loss | USF1_2 | 0.022 | 0.039 | 0.353 |
| 39 | G/T | RXRA | loss | RXRA_disc2 | 0.026 | 0.048 | 0.258 |
| 40 | G/T | MXI1 | loss | MXI1_disc2 | 0.027 | 0.049 | 0.397 |
| 41 | G/T | PAX5 | loss | PAX5_2 | 0.027 | 0.044 | 0.223 |
| 42 | G/T | GLIS2 | loss | GLIS2_2 | 0.032 | 0.045 | 0.272 |
| 43 | G/T | JDP2 | loss | JDP2_6 | 0.033 | 0.047 | 0.357 |
| 44 | G/T | Myc | loss | MA0147.2 | 0.035 | 0.045 | 0.336 |
| 45 | G/T | AP1 | loss | AP1_disc4 | 0.036 | 0.037 | 0.254 |
| 46 | G/T | MAX | loss | MAX_5 | 0.038 | 0.031 | 0.212 |
| 47 | G/T | BACH1 | loss | BACH1_1 | 0.038 | 0.038 | 0.273 |
| 48 | G/T | CREB1 | loss | CREB1_10 | 0.039 | 0.020 | 0.205 |
| 49 | G/T | ZIC1 | loss | ZIC1_2 | 0.050 | 0.046 | 0.134 |
| 50 | G/T | RAD21 | loss | RAD21_disc4 | 0.050 | 0.049 | 0.437 |
|  | T^8^ | - | | | | | |
|  | G^9^ | - | | | | | |
| 1 – reference (Ref) / alternative (SNP) allele;  2 – TF - transcription factor;  3 – binding of TF to the reference (LOSS) / alternative (GAIN) allele;  4 – binding sites with high affinity for TF;  5 – p value statistically confirming the potential gain or loss of function of the genomic region with SNP in terms of transcription factor binding;  6 – p-value for assessing the binding of TF to the Ref allele;  7 – p-value for assessing the binding of TF to the SNP allele;  8 – biological processes pathogenetically significant for IS, in which TFs that bind to the alternative allele are jointly involved (data from the Gene Ontology resource; http://geneontology.org/);  9 – biological processes pathogenetically significant for AI, in which TFs that bind to the reference allele are jointly involved (data from the Gene Ontology resource; http://geneontology.org/) | | | | | | | |
